# Tearjerkers may leave some eyes dry: Emotional reactivity to film clips from adolescence to old age

**DOI:** 10.1111/bjdp.70002

**Published:** 2025-07-07

**Authors:** Antje Rauers, Lukas Aaron Knitter, Markus Studtmann, Michaela Riediger

**Affiliations:** ^1^ Friedrich Schiller University Jena Germany; ^2^ University of Potsdam Potsdam Germany

**Keywords:** emotion induction, emotional aging, emotional reactivity, film clips

## Abstract

Emotional film clips are frequently used to induce emotions in age‐mixed samples, but past research warrants doubt that this evokes comparable effects across age groups. We investigated age differences in target‐emotion intensity and emotion specificity (the tendency to primarily respond with one target emotion rather than others), using data from a film‐rating study with 5843 individual ratings. Ninety‐nine persons from four age groups (adolescents; younger, middle‐aged and older adults) rated their emotional responses to 66 happy, fearful, angry, sad, disgusting and neutral film clips. Crossed‐random‐effects models showed differential age effects across target emotions. When age differences emerged, older adults responded more intensely and adolescents responded less intensely than other age groups. Emotional specificity was lower in older adults versus younger age groups for disgusting and neutral films, but higher for happy films. We conclude that age‐equivalent responding to emotional films may be rather the exception than the rule.

**Statement of Contribution**

**What is already known on this subject**

- Research suggests age differences in emotional responses to film clips, but the evidence was mixed.
- Integrating past results was impeded by design differences across past studies.

**What the present study adds**

- Our study is the first to compare emotional responses across 4 age groups, 6 target emotions and 66 film clips.
- For some emotions, responses were more intense and less specific in older adults and less intense in adolescents.
- Age differences varied by emotion, underlining the value of a distinct‐emotions perspective in emotional aging.

## BACKGROUND

Studies on age differences in emotional reactivity and regulation often use emotional film clips to induce emotions in the laboratory. This method presents an easy and inexpensive method for inducing emotions (Fernández‐Aguilar et al., 2019; Studtmann et al., 2009). However, developmental theories predict that it could involve systematic age effects. Assessing such potential differences is complicated by the different age groups, target emotions and specific stimuli included in past studies. To help reconcile these patterns across target emotions and age groups, we investigated differences in subjective emotional reactivity to a large number of film clips from adolescence to old age, focusing on two facets of emotional reactivity: target‐emotion intensity and specificity.

### Why should emotional responding to emotional film clips differ between age groups?

Overall, the literature suggests age differences in emotional responding to film clips. However, theoretical notions and findings are less clear regarding the exact shape of these differences, as we will review next. We focus on subjective emotional responding to film clips. Even more heterogeneity is observed across different outcomes (e.g. physiological and behavioural outcomes) or methods (e.g. pictures or relived memories).

### Emotional reactivity in older adults, compared to younger age groups

The strength and vulnerability integration model (SAVI; Charles, 2010; Charles & Luong, 2013) argues that increased physiological vulnerabilities in late adulthood may challenge emotion regulation if emotional arousal is high. This could imply that in old age, people respond more strongly if they are exposed to intense, emotionally arousing emotional stimuli that cannot be avoided – like film clips in the laboratory. Other theoretical notions warrant differential predictions across emotions. For example, differential functions of emotions across life could involve a higher preparedness for sadness in older adults and a higher preparedness for anger in young adulthood (Kunzmann & Wrosch, 2017). Other frameworks imply that older adults could be particularly motivated to savour positive over negative emotional experiences (Carstensen et al., 1999). Yet other notions emphasize core disgust as a possible exception from such age differences because it evolved to signal health threats that are relevant at any age (Rozin et al., 2008).

In essence, theoretical frameworks converge in suggesting stronger responding in older versus younger adults to fearful, sadness and happy film clips. However, they make partly diverging predictions for anger (more vs. less intense in old age) and disgust (more vs. no age differences). The available empirical evidence for these predictions is mixed, as we will review next. A summary of this evidence is provided by Table 1.

**TABLE 1:** Overview of past evidence on adult age‐group differences in emotional intensity when watching emotional film clips.

Emotion	Study	Age groups	Included number of film clips for this target emotion	Additionally included target emotions	Emotional intensity in older adults (vs. younger age groups)
Sadness	Fajula et al. (2013)	YA, OA	2	Happiness, anger, fear, disgust	+
Sadness	Haase et al. (2012)	YA, MA, OA	2	Disgust	+
Sadness	Katzorreck et al. (2022)	YA, MA, OA	4	—	+
Sadness	Katzorreck and Kunzmann (2018)	YA, MA, OA	2	—	+
Sadness	Kunzmann and Grühn (2005)	YA, OA	3	—	+
Sadness	Kunzmann and Richter (2009)	Continuous age (20–70)	2	—	+
Sadness	Mather and Ready (2021)	YA, OA	4	—	+
Sadness	Mienaltowski and Blanchard‐Fields (2005)	YA, OA	1	Happiness	+
Sadness	Seider et al. (2011)	YA, MA, OA	2	Disgust	+
Sadness	Shiota and Levenson (2009)	YA, MA, OA	1	Disgust	+
Sadness	Droulers et al. (2015)	YA, OA	3	Happiness	−
Sadness	Beaudreau et al. (2009)	YA, OA	2	Amusement, anger, fear	o
Sadness	Tsai et al. (2000)	YA, OA	1	Amusement	o
Sadness	Zempelin et al. (2021)	YA, OA	1	Happiness, anger, disgust	o
Sadness	Fernández‐Aguilar et al. (2018)	YA, OA	9	Disgust, fear, anger, amusement, tenderness	†
Anger	Beaudreau et al. (2009)	YA, OA	2	Amusement, sadness, fear	+
Anger	Charles (2005)	YA, OA	3	—	+
Anger	Fajula et al. (2013)	YA, OA	2	Happiness, fear, sadness, disgust	+
Anger	Zempelin et al. (2021)	YA, OA	1	Happiness, sadness, disgust	o
Anger	Fernández‐Aguilar et al. (2018)	YA, OA	10	Disgust, fear, sadness, amusement, tenderness	†
Fear	Fajula et al. (2013)	YA, OA	2	Happiness, anger, sadness, disgust	+
Fear	Fernández‐Aguilar et al. (2018)	YA, OA	8	Disgust, sadness, anger, amusement, tenderness	†
Disgust	Fajula et al. (2013)	YA, OA	2	Happiness, anger, fear, sadness	+
Disgust	Zempelin et al. (2021)	YA, OA	1	Happiness, sadness, anger	+
Disgust	Kunzmann et al. (2005)	YA, OA	3	—	−
Disgust	Haase et al. (2012)	YA, MA, OA	2	Sadness	o
Disgust	Scheibe and Blanchard‐Fields (2009)	YA, OA	1	—	o
Disgust	Seider et al. (2011)	YA, MA, OA	2	Sadness	o
Disgust	Shiota and Levenson (2009)	YA, MA, OA	1	Sadness	o
Disgust	Fernández‐Aguilar et al. (2018)	YA, OA	8	Fear, sadness, anger, amusement, tenderness	†
Happiness	Fajula et al. (2013)	YA, OA	2	Anger, fear, sadness, disgust	o
Happiness	Mienaltowski and Blanchard‐Fields (2005)	YA, OA	1	Sadness	o
Happiness	Droulers et al. (2015)	YA, OA	3	Sadness	o
Happiness	Zempelin et al. (2021)	YA, OA	1	Sadness, anger, disgust	o
Amusement	Tsai et al. (2000)	YA, OA	1	Sadness	o
Amusement	Beaudreau et al. (2009)	YA, OA	2	Anger, sadness, fear	o
Amusement	Fernández‐Aguilar et al. (2018)	YA, OA	7	Disgust, fear, sadness, anger, tenderness	†
Tenderness	Fernández‐Aguilar et al. (2018)	YA, OA	8	Disgust, fear, sadness, anger, amusement	†

*Note*: None of the studies included adolescents. + = more intense target‐emotion responding in older adults than younger age groups, − = less intense target‐emotion responding in older adults than younger age groups, o = no age differences. ^†^The study by Fernández‐Aguilar et al. (2018) did not investigate age differences in target‐emotion intensity but instead compared valence and arousal ratings, with mixed findings. Older versus younger adults responded with greater negativity to disgusting, fearful and angry films, with less arousal to fearful and amusing stimuli and more arousal to tender films.

Abbreviations: MA, middle‐aged adults; OA, older adults; YA, younger adults.

To summarize the evidence from Table 1, the notion of stronger responding to sad films in older adults (vs. younger age groups) is supported by the majority of studies (Fajula et al., 2013; Haase et al., 2012; Katzorreck et al., 2022; Katzorreck & Kunzmann, 2018; Kunzmann & Grühn, 2005; Kunzmann & Richter, 2009; Mather & Ready, 2021; Mienaltowski & Blanchard‐Fields, 2005; Seider et al., 2011; Shiota & Levenson, 2009), although others found the reversed pattern (Droulers et al., 2015) or no differences between age groups (Beaudreau et al., 2009; Tsai et al., 2000; Zempelin et al., 2021). Likewise, most studies found increased responding to angry film clips in older adults (Beaudreau et al., 2009; Charles, 2005; Fajula et al., 2013), with one exception finding no age differences (Zempelin et al., 2021). For highly arousing fear films, one study documents stronger emotional responses in older age adults (Fajula et al., 2013). For disgust, some findings align with theoretical notions of age‐invariant reactivity (Haase et al., 2012; Scheibe & Blanchard‐Fields, 2009; Seider et al., 2011; Shiota & Levenson, 2009), but others found more intense (Fajula et al., 2013; Zempelin et al., 2021) or less intense (Kunzmann et al., 2005) responding in older (vs. younger) adults. Finally, no age differences were found in responding to happy (Droulers et al., 2015; Fajula et al., 2013; Mienaltowski & Blanchard‐Fields, 2005; Zempelin et al., 2021) or amusing (Beaudreau et al., 2009; Tsai et al., 2000) film clips. In sum, age‐related differences in emotional responding seem to depend on the target emotion. The question of age differences in subjective responding to film clips should therefore best be studied considering a range of target emotions. Only a few studies have done this so far, with mixed results. One study found stronger responding to four film clips (sadness, anger, fear and disgust) in older adults, compared with younger adults, but no age differences in two other film clips (neutral and happy, Fajula et al., 2013). Another study found stronger responding in older adults (vs. younger adults) to an anger film, but a reversed pattern for an amusing film and no age differences for a sad and fearful film (Beaudreau et al., 2009).^1^ In these studies, each emotion was only represented by one or two film clips. This delimits the generalizability of these findings because responses may differ across film stimuli (Jenkins & Andrewes, 2012; Katzorreck & Kunzmann, 2018; Kunzmann & Grühn, 2005; also see pre‐study for Labuschagne et al., 2020). One study with younger and older adults used up to five film clips per emotion (Fernández‐Aguilar et al., 2018), but looked at valence and arousal, instead of specific emotional responses. Its results were mixed and partly incompatible with findings from distinct‐emotion approaches. In the present study, we adopted the distinct‐emotions approach that is predominant in the literature, allowing us to examine potential differences between emotions. In sum, the literature suggests there may be age differences in emotional responding, but theories and findings diverge in predicting the exact form of these differences. Across target emotions, theories and findings lean towards more pronounced emotional responding in old age. This led us to hypothesize that emotional responding to standardized emotional film clips would be stronger in late adulthood, compared to younger age groups (H1). The marked conceptual and empirical heterogeneity regarding possible emotion‐specific age effects did not warrant any hypotheses regarding interactions of age with target emotion. We therefore investigated such interactions in an exploratory fashion.

## EMOTIONAL REACTIVITY IN ADOLESCENCE, COMPARED WITH YOUNGER ADULTS

Our second prediction pertained to higher emotional reactivity in adolescents, compared with younger adults. Adolescence, the transition period between childhood and adulthood, is a time of marked emotional instability (Bailen et al., 2019; Griffith et al., 2021) and pronounced reactivity to emotional stimuli and stressors, particularly those involving rewards or social information (Crone & Konijn, 2018; Spear, 2011). Reasons involve a temporal maturational imbalance between limbic activation and executive control during adolescence (for overviews, see Casey, 2015; Shulman et al., 2016). This suggests that adolescents may be particularly responsive to emotional film clips. One study indeed found that adolescents, compared to younger adults, responded more strongly to two negative film clips (Herry et al., 2019). However, no empirical study to our knowledge has compared adolescents with middle‐aged or older adults. We hypothesized that adolescents would respond more strongly to emotional film clips, compared to younger adults (H2). Other age‐group comparisons and emotion‐specific effects were investigated exploratorily.

### Emotion specificity across age groups

Researchers typically seek to induce specific, distinct target emotions. However, people may experience alternative emotions or blends of emotions. Evidence is accumulating that both tendencies vary across age groups. We therefore also investigated the specificity of participants' responses, defined as a high degree of experiencing a discrete target emotion over alternative emotions. Theoretical notions suggest that age‐related increases in life experience result in more complex emotional responses (Labouvie‐Vief, 2003). In line with this notion, older adults are more likely than younger adults to experience multiple emotions (e.g. Charles, 2005; Kliegel et al., 2007; Mather & Ready, 2021) and non‐target emotions (e.g. Haase et al., 2012) when watching emotional film clips. We therefore predicted that emotion specificity—the tendency to predominantly experience the target emotion over alternative emotions—would be lower in older adults than in younger age groups (H3).

### Objective of the present study and hypotheses

The evidence on age differences in subjective reactivity to emotional film clips is inconclusive and mostly pertains to selected age groups, target emotions and individual films. This impedes the integration of past findings, which motivated the present study. We investigated age differences in the intensity and specificity of subjective emotional responses to film clips in four age groups: adolescents, younger adults, middle‐aged adults and older adults (overall age span: 12–80 years), using five different target emotions (as well as neutral control stimuli) and eleven films per emotion on average. We hypothesized (H1) higher emotional reactivity in older adults, compared to younger age groups, (H2) higher emotional reactivity in adolescents, compared to younger adults and (H3) lower specificity in older adults' emotional responses, compared to younger age groups.

## METHOD

### Transparency and openness

We report how we determined our sample size of observations, all data exclusions (if any), all manipulations and all measures in the study and we follow JARS (Kazak, 2018). The original research data, research materials, model equations and analysis code are documented at https://osf.io/jyzqa/?view_only=f3051fa0ed434daab9fb91ba22ddc9f3. The data were analysed using SPSS, Version 26 and R, version 4.1.0 (R Core Team, 2022) in the integrated development environment RStudio. This study's design and its analysis were not pre‐registered.

### Compliance with ethical standards

The Max Planck Institute for Human Development Ethics Committee had approved all stimuli and the study procedure before data collection. Participants provided written consent for participating. Parental consent was obtained for adolescents. Participants were informed that they could quit the study at any time, but no participant made use of this option.

### Participants

The purpose of the present study was to investigate age differences in emotional intensity and emotion specificity. To this end, we reanalysed data from a previous data collection that was initially conducted to obtain an empirical basis for selecting film clips to induce emotions in different age groups. This yielded a database of *N* = 5843 subjective ratings from 99 people on 66 film clips for six target emotions. For the present study, we used these data to investigate age differences in emotional intensity and emotion specificity. Our sample was recruited from the urban area of Berlin, Germany, using our subject pool. Each participant rated up to 66 film clips, yielding an effective observation sample size of *N* = 5843. The design aimed to maximize the effective observation sample size to the extent that is possible without overburdening participants. The data were collected from May to July of 2011. There were four age groups: We included 25 younger adults (aged 20–30 years) and 25 older adults (aged 70–80 years) to cover the age range typically included in studies using student and community‐dwelling older adult samples. Additionally, we included 28 adolescents and 21 middle‐aged adults. The age ranges for these two groups (12–16 years and 45–55 years, respectively) were chosen to maximize the age distance to younger and older adults, respectively. In the total sample, 47.5% identified as female and 46% as male (adolescents: 46% female, younger adults: 52% female; middle‐aged adults: 48% female; older adults: 44% female). Thirty‐nine percent of the participants had graduated from high school or a higher educational institution (none of the adolescents, 76% of the younger adults, 57% of the middle‐aged adults and 32% of the older adults, respectively). All adolescents and younger adults were unmarried. Among the middle‐aged adults, 24% were unmarried, 43% were married and 33% were divorced. Among the older adults, 8% were unmarried, 40% were married, 16% were divorced and 36% were widowed. Race was not assessed. Given possible age differences by target emotion and individual film clips, our goal was to include a large number of target emotions and film clips. We asked each participant to rate 43 (adolescents) or 66 (the other age groups) film clips across 2 (adolescents) or 3 (the other age groups) sessions. Some of the film clips were excluded for adolescents due to youth protection. This yielded a total sample of *N* = 5890 observations (i.e. target‐emotion ratings; *n* = 1204 observations for adolescents and *n* = 4686 observations for the other age groups).

### Stimuli

To put our hypotheses of age differences in emotional responding to a conservative test, we aimed at using stimuli that would intensely induce various distinct target emotions with high specificity across different age groups. Our protocol for selecting the films is described next. The film clips used in our study are described in section S1 in the Data S1. Their presentation complied with copyright regulations.

With the aim to include approximately 10 stimuli per target emotion, we screened previous publications, movies and public video platforms for film clips that we expected to intensely, and specifically, elicit one out of five target emotions: sadness, anger, fear, happiness and disgust. Our selection heuristic was based on propositions of appraisal theory (Lazarus et al., 1970; Scherer et al., 2001): Films showing a protagonist who experienced something pleasant or desirable were chosen for their potential to elicit happiness. Films implying pending danger or loss of control were selected as candidates for fear films. Clips showing injustice or violations of social rules were selected for their potential to elicit anger. Films dealing with severe losses and little chance of amendment were included to elicit sadness. Films showing unhygienic and potentially health‐threatening scenes were selected as disgusting films. Finally, we included neutral films, among them some own recordings (e.g. pedestrians passing by). This was done to control for acquiescence (i.e. the tendency to endorse an item, which may increase with adult age; e.g. Lechner & Rammstedt, 2015). We submitted this initial pool to internal piloting among nine members of our research team (22% male; six younger adults aged 20–30 years, two adults aged 30–40 years and one person aged 40–50 years). This team provided two to six ratings per video clip regarding the assumed potential to strongly induce the target emotion across age groups (0 = not at all suitable; 5 = very suitable). In these initial ratings (*M* = 3.69, *SD* = 0.81, range: 1.33–5.00), overall agreement was low (ICCs from .23 for clips with five ratings to .36 for clips with two ratings). All clips were therefore reviewed in consensus meetings. We first excluded film clips with ethical concerns, a length exceeding 3 min, low video quality, and obvious potential of lower salience or accessibility for some of the age groups (e.g. films with outdated language, futuristic settings or very specific pop cultural references). Next, we aimed to include films with at least four ratings resulting in high (*M* = 4 out of 5 points) and homogeneous suitability ratings (maximum *SD* = 1). Films that had been used in past studies (e.g. ‘cry for freedom’) were included with a threshold rating of 3 and a maximum standard deviation of 1.5. The top selection comprised *N* = 66 film clips for adults (nine stimuli for happiness, 12 for fear, 10 for anger, 14 for sadness, 11 for disgust and 10 neutral stimuli). Two independent raters coded the gender and age group of the protagonists in the film clips (*ĸ* = 0.83) and a third coding was obtained for deviating codes. The films included male (77%) and female (59%) protagonists from different age groups, namely babies or children (0–11 years; 20% of the films), adolescents (12–19 years; 11%), younger adults (20–30 years; 29%), middle‐aged adults (30–59 years; 65%) and older adults (older than 60 years of age; 18%). The average length of the film clips was about 1 min (*M* = 57.82 s, *SD* = 38.33).

*N* = 43 of these stimuli were also appropriate for presentation to underage participants and were shown to the adolescent participants (nine stimuli for happiness, none for fear, six for anger, 10 for sadness, eight for disgust and 10 neutral stimuli).^2^

We asked participants after each clip whether they had seen it before. This was the case for 14% of the film clips (*SD* = 15), with no significant age differences (*F*(3) = 2.22, *p* = .31).

### Design and implementation

Participants watched 22 films in each testing session. Adults watched 66 film clips over the course of three test sessions (with 22 films in each session) and adolescents watched 22 and 21 film clips, respectively, across two sessions. We randomly assigned films to sessions, with the constraints that each of the five target emotions and neutral films should be equally represented within each session, and that films of the same target emotion were never presented twice in a row. The assignment of films to sessions was fixed across participants, but the order of the sessions varied between persons and was counterbalanced within each age group. Within each session, the order of the stimuli was randomized for each person. After each stimulus, participants were presented with questions on the computer screen regarding their emotional experiences during the film clip. They responded to these questions by using the numbers on the computer keyboard. Stimuli presentation and recording of participants' responses were implemented in DMDX (Forster & Forster, 2003). Film clips, questions and response options were presented on 17‐inch computer screens (full‐screen view) with a resolution of 1280 × 1024 pixels. The study was conducted in small groups of up to six persons. Participants were assigned partitioned testing nooks, were seated approximately 2 feet apart from their monitor and wore headphones with individually adjustable volumes.

### Procedure

Demographic information was assessed at the beginning of the first session. Participants were then told, ‘In the following, you will see several short film clips. We would like to know how you perceive these films and which emotions you experience while watching them. After each film, you will be asked a few questions that can be answered using the computer keyboard’. After each film clip, participants provided ratings on their emotional experiences during the films. First, they were asked, ‘How strongly did you feel happiness?’ (0 = *not at all* to 6 = *very strongly*). This question was then repeated for disgust, fear, sadness, anger, amusement, shame, surprise and neutral. Surprise, amusement and shame were included as additional potential rival target emotions. Participants were instructed to rate the peak intensity for these nine emotional states throughout the film clip. These ratings provided the basis for two outcome measures: First, we were interested in participants' peak intensity ratings on the target emotion (i.e. the emotion that a film clip was specifically pre‐selected to induce). Second, we were interested in the specificity of participants' responses, operationalized as the distance from participants' target‐emotion ratings from ratings on alternative emotions (i.e. the non‐target emotions).

Time conflicts caused one young man to drop out of the study after the first testing session, so he only rated 26 films. Two individuals' ratings for one individual film were missing due to irregularities in starting the first film. For another participant, one individual disgust rating for one film was set to missing after the participant requested this.

### Measures

#### Target‐emotion intensity

Intensity measures how strongly participants responded on the target emotion. It describes a given participant's rating on the target emotion for a given particular film clip (e.g. their sadness rating after watching a sad film).

#### Specificity

Specificity addresses how strongly an emotional reaction was confined to the emotion that the respective film clip targeted to elicit, relative to other, non‐target emotions. This variable was calculated as the difference of the target‐emotion rating of a given person for a given film clip, minus the highest non‐target rating provided by this person for this film clip. The higher this score is, the more specifically a person's response focused on the target emotion (compared to other emotions). Specificity scores greater than zero indicate that the target emotion was the highest‐rated emotion and scores smaller than zero indicate that at least one emotion other than the target emotion was endorsed more strongly. Our operationalization of emotion specificity followed the rationale to obtain a conservative measure: it was sensitive to even minor variations that could be caused by as little as one single rival emotion.

### Statistical analyses

We used crossed‐random effects models (Baayen et al., 2008; Judd et al., 2017) to account for statistical interdependencies within participants (repeated ratings of a given individual across film stimuli) and within film stimuli (repeated ratings of a given stimulus across participants). Using the lmerTest package (Bates et al., 2015), we predicted participants' ratings of the subjective intensity of the emotion targeted by a given film clip (H1 and H2) or their rating specificity (H3) by including the target emotion (i.e. the emotion that the film was intended to elicit; dummy coded with the neutral condition as reference), participants age group (dummy coded with varying reference groups across models, see below) and their interaction as fixed effects, using restricted maximum likelihood estimation (REML). Interactions of age group X emotion estimate if the difference in emotional reactivity (i.e. intensity and specificity) between the neutral control condition and any given target emotion was moderated by age group. To follow up on individual contrasts, we repeated the same model with recoded dummy codes for emotion and age group. That is, we did not repeatedly test our hypothesis. Instead, we tested our hypothesis once and then ran variants of the same model with altered dummy codes to understand the exact nature of the effects. This allowed us to avoid alpha error accumulation as would be implied by repeated testing.

Random effects were included to account for the statistical interdependencies in the data. Likelihood ratio tests were used to test whether including these effects in the model was meaningful, which we assumed to be the case when removing a given random effect yielded a model fit that was significantly worse than the full model. Both for the models predicting target‐emotion intensity and specificity, the best‐fitting, non‐singular model included three random components: a random intercept for film clips, to model the variance in ratings due to differences between the film clips; a random intercept for persons, to account for the variance in ratings between participants and a random slope for participants' responses on each of the target emotions. The latter accounts for individual rating patterns (e.g. some individuals may have been particularly susceptible to sad film clips, while others may have responded more strongly to anger‐inducing film clips). The equations for the final models used for hypothesis testing are shown in Section S2 in the Data S1, and the model development is documented in Section S3. To determine the overall variance explained by these models, we calculated the marginal and conditional *R* ^2^ according to Nakagawa and colleagues (Nakagawa et al., 2017).

## RESULTS

### Age differences in target‐emotion intensity (H1 and H2)

**FIGURE 1:**
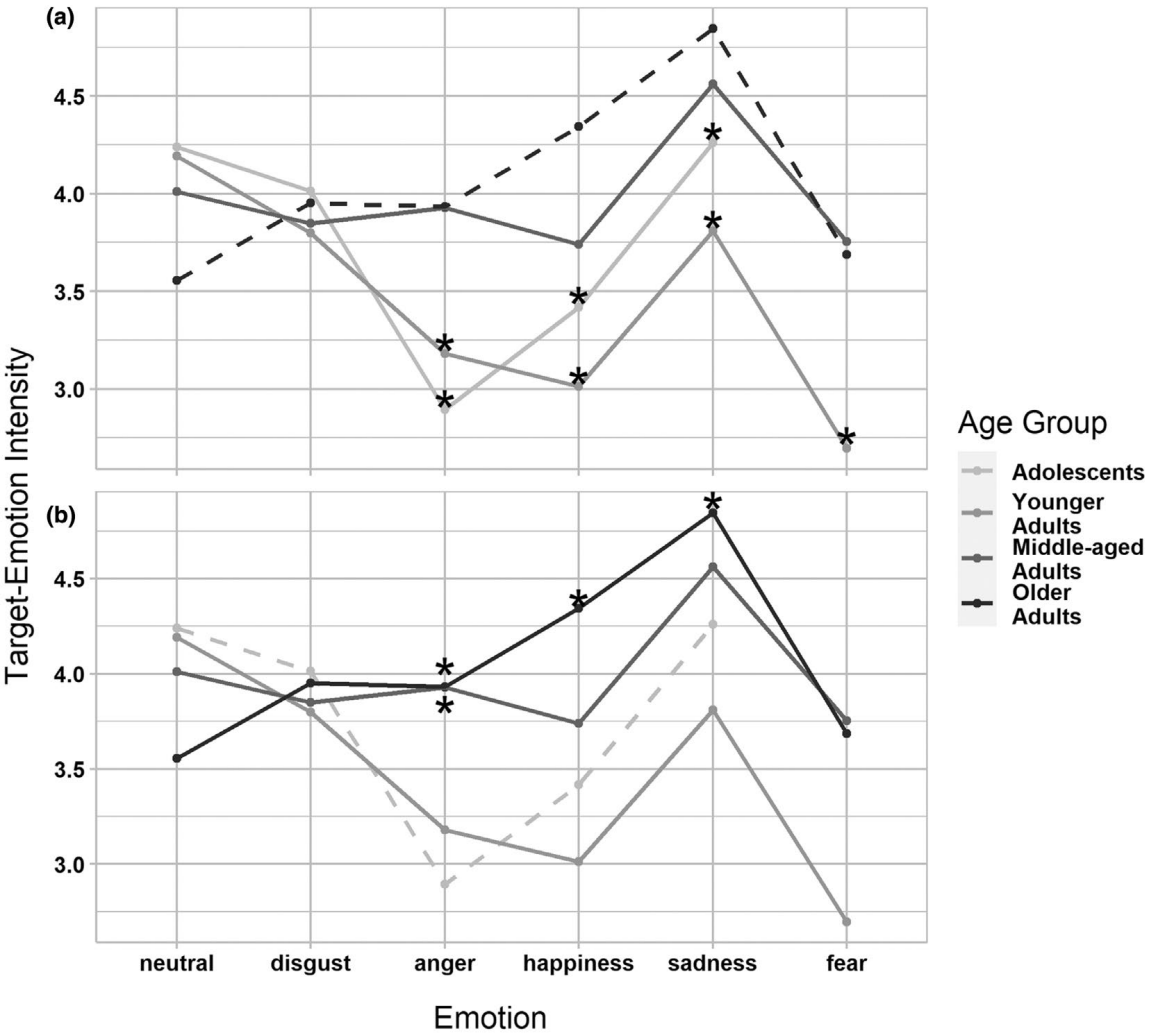
Estimated means in intensity ratings by age group and emotion. Estimated mean intensity ratings (theoretical range: 0–6) by emotion and age group, with older adults (dashed black line) and neutral films as reference group (panel a) or adolescents (grey dashed line) and neutral films as reference groups (panel b), respectively. Estimates were obtained from crossed random effects models with *N* = 99 persons and *N* = 66 film clips (*N* = 5843 observations). For each target emotion, asterisks (*) indicate a significant difference (*p* < .05 and CI excluded zero) from the estimated mean of the reference group. This significant difference pertains to the fixed effect parameter estimate for the interaction of that age group × emotion. The parameter estimates that provided the basis for these panels are documented in Section S3.1 in Data S1. Adolescent participants did not watch any fear videos for ethical reasons.

In the model predicting target‐emotion intensity, the marginal *R* ^2^ (i.e. the proportion of variance explained by the fixed effects alone) was .061, and the conditional *R* ^2^ (i.e. the proportion of the variance explained by both the fixed and random effects) was .477. That is, the model explained 48% of the variance in the individual ratings. Differences between persons and film clips accounted for 42% of the variance. Six percent of the variance was explained by age group and target emotion, which indicates small effects. These effects are illustrated in the model results as depicted in Figure 1. It shows the estimated mean ratings of target emotion by age group and emotion and highlights significant differences between age groups. Model predictions are shown for model variants using older adults and adolescents as reference groups to address H1 and H2, respectively (for documentation of all parameter estimates from these models, see Section S3.1 in Data S1).

In hypothesis 1, we had predicted higher emotional reactivity in older adults, compared to the younger age groups. In line with this hypothesis, older adults responded more strongly than younger adults and adolescents to sad, angry and happy films. They also responded more strongly than younger adults to fearful films (for which no ratings by adolescents were available). In contrast, there were no differences between older and middle‐aged adults in this regard. In hypothesis 2, we had predicted higher emotional reactivity in adolescents, compared with younger adults. This hypothesis was not confirmed. Apart from the abovementioned differences compared with older adults, adolescents were less reactive to anger films if compared with middle‐aged adults. There were no differences between any of the four age groups in disgust reactivity and no age differences in people's reactivity to the neutral film clips that served as the control condition.^3^ We repeated the analyses with younger and middle‐aged adults as reference groups, respectively. There were no additional age‐group differences involving these groups. That is, any age difference observed in target‐emotion intensity involved comparisons with either older adults or adolescents (Figure 1 and Section S3.1 in Data S1).

A post‐hoc simulation‐based power analysis with the simR package (Green & Macleod, 2016; 500 simulations) suggested a high power for detecting interaction effects of age group and emotion (99.40%; 95% CI: [98.26, 99.88]), but a limited power for detecting a main effect of age group (43.00%; 95% CI: [38.61, 47.47]).

In summary, our prediction of higher reactivity in older compared to younger age groups was partially supported, but not for all age group comparisons and all emotions. Our second hypothesis of stronger reactivity in adolescents, compared to younger adults, was not confirmed. Instead, adolescents responded less strongly than some age groups, and again, this was not true across all emotions.

### Age differences in emotional specificity (H3)

**FIGURE 2:**
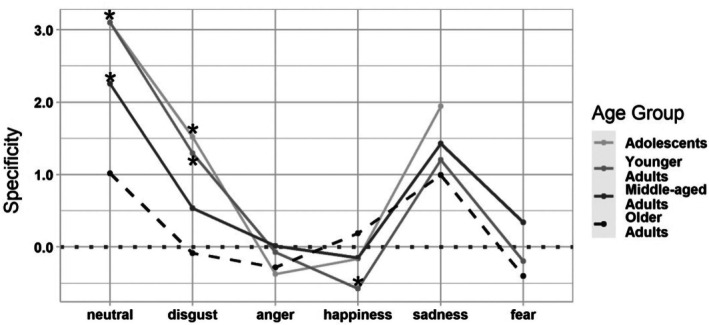
Estimated mean emotional specificity by age group and emotion. The figure illustrates the estimated mean specificity of ratings (theoretical range: −6 to 6) by emotion and age group. Estimates were obtained from crossed random effects models with *N* = 99 persons and *N* = 66 film clips (*N* = 5843 observations). Higher scores indicate more specific reactivity on the target emotion (over other emotions). Estimates above zero (above the dotted vertical line) indicate that on average, people rated the target emotion as highest (and provided less intense ratings for all other emotions). Estimates below zero indicate that on average, people rated at least one alternative emotion higher than the target emotion. Note that these alternative emotions varied across participants and film clips, however. On average, all of the specific rival emotions were rated lower than the target emotion. Age‐group differences in specificity across emotions were tested using dummy codes for age group and emotion, as well as their interaction, as predictors. Following up on the significant interaction effect of Age Group X Emotion, we repeated the model, swapping the target emotion in question as the reference category, while maintaining older adults as the reference category throughout. Main effects of age in these follow‐up models (*p*s < .05 and CI excluded zero for interactions of emotion X age group) emerged for neutral, disgust and happiness. In the figure, asterisks (*) indicate a significant difference from the reference group of older adults. The estimated means for the figure are documented in Section S3.2 in Data S1. Adolescent participants did not watch any fear videos for ethical reasons.

We repeated the models using emotion specificity as the dependent variable. The marginal *R* ^2^ (i.e. the proportion of variance explained by the fixed effects alone) was .128, and the conditional *R* ^2^ (i.e. the proportion of the variance explained by both the fixed and random effects) was .434. That is, the model explained 43% of the variance in the individual ratings. Differences between persons and film clips accounted for 31% of the variance. Thirteen percent of the variance was explained by age group and target emotion, indicating small effects. These are illustrated in the model results as depicted in Figure 2. This figure also shows that, while the films were quite successful overall in inducing strong emotional experiences, the specificity of participants' responses was mixed. Estimates below zero indicate that on average, people rated at least one alternative emotion higher than the target emotion. These alternative emotions varied across participants and film clips, however. Therefore, specificity estimates below zero do not imply that any specific rival emotion was rated more intensely overall compared with the target emotion. In fact, average intensity ratings for the target emotions were higher overall compared with all other emotions, and this was true across all emotions and all age groups. Therefore, even target emotions with specificity estimates lower than zero still received the highest intensity ratings on average across participants and film clips. Hypothesis 3 predicting lower specificity in older adults was supported for most emotions. However, the predicted age differences were not consistent across all emotions. These differential effects were tested using cross‐random effects models with neutral and older adults as reference categories. The individual parameter estimates are documented in Section S3.2 in Data S1. For neutral, the estimated means for all age groups were above zero, indicating that neutral films were indeed perceived as mostly neutral on average. However, the main effects for adolescents, younger adults and middle‐aged adults were significant compared to the reference group of older adults (*p*s < .05 and CI excluded 0). This indicates that the specificity of older participants' ratings of neutral film clips was significantly lower compared with any of the three other age groups.

A comparable pattern emerged for disgusting film clips. There were no significant age group interactions with disgust in our model (with older adults and neutral emotions as reference groups; all *p*s > .05 and CIs included 0). This indicates that age differences in disgust specificity did not differ significantly from the age differences that we observed for specificity regarding neutral films. For all other target emotions – anger, happiness, sadness and fear – significant interaction effects of age group X target emotion emerged (*p*s < .05 and CIs excluded 0). This indicates that the age differences in specificity were significantly smaller or reversed for these emotions, compared with the age differences in the neutral control stimuli. To follow up on these interactions, we ran five additional models. Older adults remained the reference group in all of them, but we recoded the dummy codes for emotion such that each target emotion served as the reference category in one of the models. In these follow‐up analyses, significant main age effects indicate age differences in specificity for the emotion that serves as the reference category (see results table in Section S3.2 of Data S1). Figure 2 illustrates the effects: For neutral films, older adults' specificity was significantly lower compared to any of the other age groups. For disgusting films, older adults' specificity was also significantly lower compared to adolescents and younger adults. Older adults' specificity means also appeared to be comparatively low for sadness, fear and anger, but these age differences did not reach significance. Yet another pattern emerged for happy films: Here, older adults showed the most specific happiness reactivity of all age groups, with differences reaching significance when compared to younger adults' specificity. All four age groups converged in rating amusement and neutral as the most common additional emotions when watching happy films.

We again conducted a post‐hoc simulation‐based power analysis with the simR package (Green & Macleod, 2016; 500 simulations). This suggested a 100% power to detect interaction effects of age group and emotion [95% CI: 99.26, 100] and a power of 26.20% [95% CI: 22.40, 30.29] to detect a main effect of age group.

In sum, the specificity of emotional responses varied by target emotion, as did age differences in specificity. When age differences emerged, older adults' responses were the least specific of all age groups, except for the emotion of happiness, where this pattern was reversed.

### Explorative analyses: Effects across individual film clips

**FIGURE 3:**
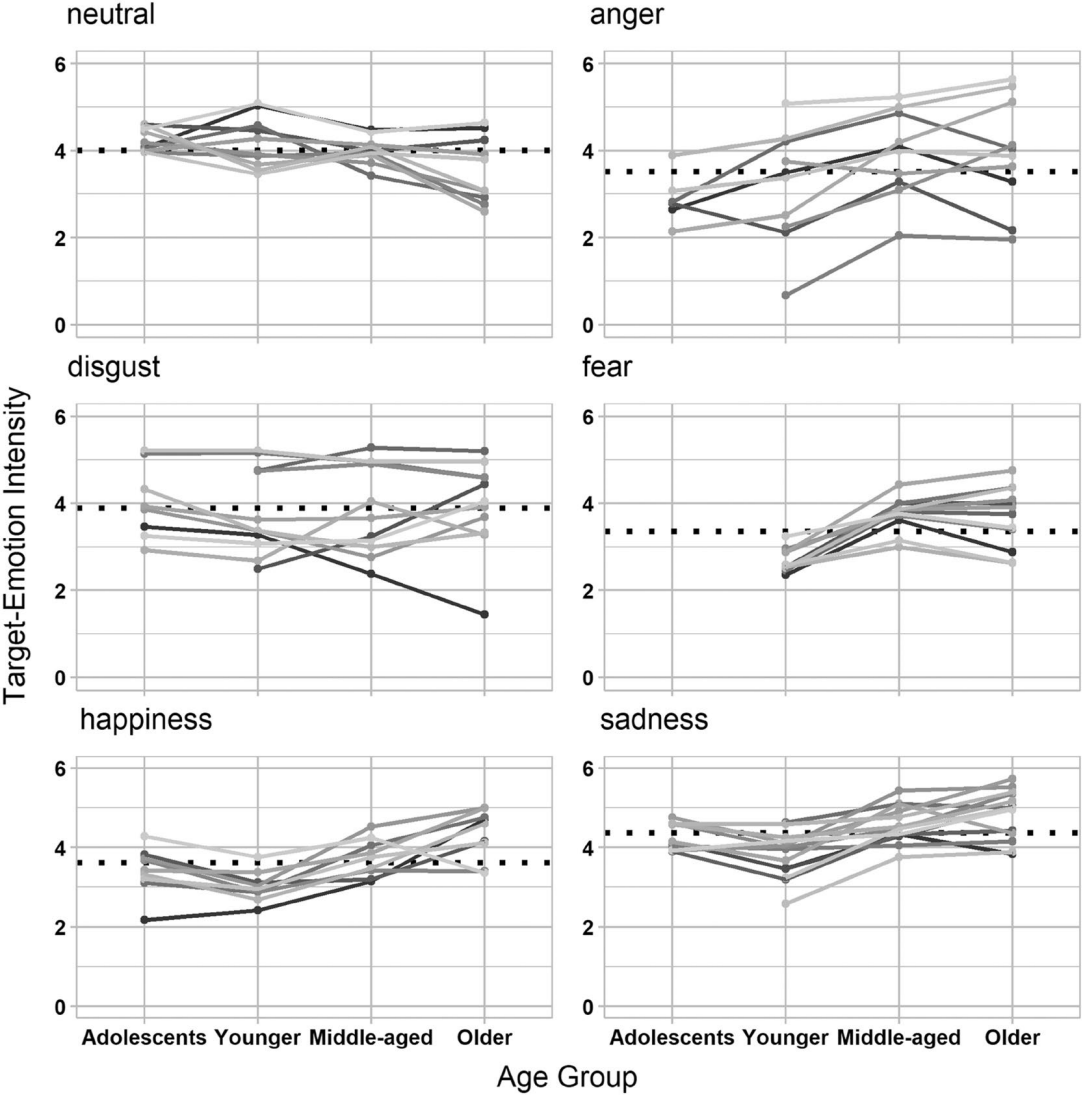
Observed mean target‐emotion ratings by film clips and age groups. Each panel (for five different target emotions and neutral films) shows the observed mean target‐emotion ratings by film clip and age group (theoretical range: 0–6). Means that pertain to the same film stimulus are connected by a solid line. The dotted vertical line indicates the observed grand mean across age groups and film clips for that emotion. While there were differences in the overall intensity across individual film clips, the age‐related pattern was predominantly homogeneous across film clips. As an exception, age differences in intensity varied across individual film clips for anger. Additionally, two happiness (out of nine) and one disgust film (out of eleven) deviated from the overall age‐related pattern.

Finally, we explored the role of individual stimuli for the observed pattern of results at the example of target‐emotion intensity ratings. We performed separate multilevel analyses by target emotion and requested a random intercept for participants to account for overall differences in ratings across persons (see Section S3.3 in Data S1 for the development and results of these models). We then predicted the target‐emotion rating with *k*−1 effect codes for each film clip (effect coded; *k* being the number as film clips), age group (effect coded with older adults as reference group) and the interaction of film clips and age groups. Figure 3 illustrates the direction of the effects by showing the observed mean target‐emotion intensity rating separately by target emotion, age group and films.

We found that, for all five emotions as well as neutral films, target‐emotion ratings varied between individual film clips. These overall differences between individual film clips are reflected in significant fixed main effects for these films (see results tables in Section S3.3 in Data S1). While some effects also varied by age group, the age‐related pattern was rather consistent. Interaction effects of age group × individual film clip emerged for anger, happiness, and disgust. For anger, this interaction effect derived from a differential age‐related pattern across individual film clips. In contrast, differences between film stimuli were the exception for disgust and joyful films: There was only one disgust film and two joyful film clips that deviated from the predominant pattern for these emotions (i.e. age‐invariant ratings for disgust and more intense joy ratings in older, compared to younger age groups). We also found that fear films elicited increasingly more variable fear responses across stimuli with increasing adult age, reflected as a higher proportion of variance explained by the random intercept for participants when predicting fear, compared to the models for other emotions. Of note, this did not qualify the overall age effect for fear, which was consistently found across fear clips.

In essence, the pattern on the level of individual film clips reflected more homogeneity than differences between films. However, the differences that we did observe emphasize the need for careful selection of video stimuli in emotion‐induction studies. We publish the original ratings for all individual film clips and age groups in the supplement, and researchers may refer to these data in selecting film stimuli for future studies. We refrain from recommending any individual film clips here because selection criteria can differ across studies. For example, study designs may require that this selection is optimized for two age groups, while others require equal consideration of selection criteria for three or four age groups. Study requirements may also place varying emphasis on intensity, specificity or topic of the film clips. The data in the supplement can be used to maximize the fit with these varying demands.

## DISCUSSION

We investigated age differences in subjective emotional reactivity to emotional film clips in the laboratory. To help bridge the inconclusive evidence from past studies with manifold design differences, we extended the number of age groups, target emotions and film clips per emotion, compared to past studies. As hypothesized, we found age differences in emotional reactivity. These pertained to two aspects of participants' responses: the intensity and specificity of participants' responses. Regarding intensity, and in line with our predictions, older adults' average reactivity to sad, angry, happy and fearful stimuli was more intense than younger adults' and adolescents'. This pattern aligns with theoretical notions of increased responding to intense emotional stimuli in late adulthood (Charles, 2010; Charles & Luong, 2013). It also converges with the majority of past findings for fear (Fajula et al., 2013; Fernández‐Aguilar et al., 2018), anger (Beaudreau et al., 2009; Charles, 2005; Fajula et al., 2013; Fernández‐Aguilar et al., 2018) and sadness (Fajula et al., 2013; Haase et al., 2012; Katzorreck et al., 2022; Katzorreck & Kunzmann, 2018; Kunzmann & Grühn, 2005; Kunzmann & Richter, 2009; Mather & Ready, 2021; Mienaltowski & Blanchard‐Fields, 2005; Shiota & Levenson, 2009). Age effects in intensity varied across film clips for anger but were more homogeneous for other emotions (except one disgust film and two happiness films). Our finding of stronger happiness reactivity in older versus younger adults seems compatible with theoretical notions (Carstensen et al., 1999), but is at odds with past evidence (Fajula et al., 2013; Fernández‐Aguilar et al., 2018; Mienaltowski & Blanchard‐Fields, 2005; Schweizer et al., 2019; Zempelin et al., 2021). A reason for this may be that we deliberately excluded bitter‐sweet (e.g. Fernández‐Aguilar et al., 2018) or humorous (Fajula et al., 2013; Mienaltowski & Blanchard‐Fields, 2005; Zempelin et al., 2021) stimuli as happy films. Our second hypothesis of higher reactivity in adolescents was not supported. Instead, reactivity to sad, angry and happy films was lower in adolescents than in older age groups. These findings are at odds with a previous study (Herry et al., 2019) The reasons for adolescents' lower reactivity remain speculative at this point. Possibly, film clips do not effectively address social information that has been linked to adolescent reactivity (Crone & Konijn, 2018; Spear, 2011). These cues may be too infrequent when passively watching films, even if they are highly emotional.

Disgust was the only emotion without age differences in target‐emotion intensity. This aligns with theoretical notions (Rozin et al., 2008) and most of the available evidence (Haase et al., 2012; Scheibe & Blanchard‐Fields, 2009; Seider et al., 2011; Shiota & Levenson, 2009). Interestingly, disgust also stands out in emotion recognition. This convergent pattern could be related to brain regions such as the basal ganglia, which are implicated in processing disgust and are relatively spared by normal brain ageing (Cortes et al., 2021; Gonçalves et al., 2018; Henry et al., 2008). To summarize, age differences varied across emotions. Whenever they emerged, they were in the direction of more intense responding in older adults, and less intense responding in adolescents. The emotion‐differential findings are mostly compatible with the literature, but we had not predicted the exact nature of this differential pattern. This warrants replication, especially in light of the post‐hoc power analyses that showed sufficient power to detect the reported interaction effect of age group and emotion, but not the predicted main age effect.

### Age differences in emotional specificity

As was the case for intensity, the specificity of participants' responses was qualified by age and target emotion. In line with our predictions, older adults showed the least specific responses of all age groups for disgusting and neutral films. Differences were in the same direction for anger, sadness and fear, but did not reach significance. This pattern aligns with the notion of more associative semantic processing in old age (Labouvie‐Vief, 2003) and with evidence linking nontarget reactivity to normative (Stephens et al., 2023) and neurodegenerative (Chen et al., 2017) age‐related changes. Both normative and pathological neurodegenerative changes become more likely with aging, which could have contributed to the present findings. Our results replicate earlier findings (Charles, 2005; Haase et al., 2012; Kliegel et al., 2007; Mather & Ready, 2021) and extend them by offering a discrete‐emotions perspective. Interestingly, the pattern was reversed for happy films. Here, specificity was higher in older, compared to younger adults. This could be related to older adults' valuation of positive emotions as unequivocally positive (Cohrdes et al., 2017; Riediger et al., 2014).

### Limitations and conclusions

To our knowledge, the present investigation is the first to use a large pool of different film clips to investigate emotional responding across five target emotions in four age groups. Past findings yielded inconsistent age effects across studies, but past studies typically focused on a few target emotions, age groups and film stimuli. This raised the question of whether design differences or differences between individual film clips caused the inconsistent patterns. However, differential effects also emerged in the present study, within the same sample and using a large number of individual film stimuli. Age effects varied across emotions but were largely homogeneous across individual film clips (with a notable exception for anger that also evoked less specific responses overall, across age groups). The emotion‐differential effects point to the possibility that heterogeneous patterns in past studies were not entirely due to differences in designs. Instead, this may point to genuine differential age effects across target emotions. Our results underline the value of considering differential age effects for distinct emotions in emotional aging (Haase et al., 2012; Kunzmann et al., 2014; Kunzmann et al., 2017). In essence, the question of age differences in emotional responding to film clips may best be answered separately for different emotions. Our results are limited to subjective responding and may not converge with physiological or behavioural responding (Fernández‐Aguilar et al., 2020; Shiota & Levenson, 2009; Tsai et al., 2000). They may also not generalize to other emotion‐induction methods or experiences in daily life. Finally, these cross‐sectional data cannot discern age from cohort effects. Notwithstanding these open questions, our study cautions against the notion of age‐equivalent responding to film clips: It may rather be an exception than the rule. This should be considered when planning emotion‐induction studies with age‐mixed samples.

## AUTHOR CONTRIBUTIONS

**Antje Rauers:** Conceptualization; writing – original draft; data curation. **Lukas Aaron Knitter:** Formal analysis; writing – review and editing; visualization; methodology. **Markus Studtmann:** Writing – review and editing; conceptualization; data curation. **Michaela Riediger:** Conceptualization; writing – review and editing.

## CONFLICT OF INTEREST STATEMENT

The authors have no known conflicts of interest to disclose.

## Supporting information


Data S1

## Data Availability

The original research data, research materials, model equations, and analysis code are available at https://osf.io/jyzqa/?view_only=f3051fa0ed434daab9fb91ba22ddc9f3.

## Footnotes

^1^
Yet another study (Jenkins & Andrewes, 2012) performed repeated t‐tests for individual clips but did not test age differences within or across target emotions.

^2^
Clips from movies were only shown to adolescent participants if the entire movie had been rated appropriate for adolescents aged 12 or older by the German Film Industry (German FSK 12). Due to these regulations, no fear stimuli were included for adolescents. For the same reason, two anger films, three disgusting films, and one sadness film were excluded for adolescents. After the exclusion of these clips, we considered two additional anger stimuli and three additional sadness stimuli as inappropriate for adolescents and excluded them for this age group. During the testing sessions, participants also watched and rated 12 amusing films. These ratings were collected as part of a separate investigation for which we investigated responses to amusing stimuli across two different lifespan samples.

^3^
Like the age comparisons for any of the five target emotions, this conclusion for neutral films derives from considering four different models with recoded dummy codes for each age group. However, neutral served as control condition in all models. The conclusion that there were no age differences in responding to neutral film clips is therefore based on the fixed‐effects estimates for the main effect of the age groups across these four models. The results of all models with all estimates are included as supplemental material.
