# Supplementary material for: Tearjerkers may leave some eyes dry: Emotional reactivity to film clips from adolescence to old age
Source: Br J Dev Psychol. 2025 Jul 7;44(1):33–51. doi: 10.1111/bjdp.70002 (PMC12884362; doi:10.1111/bjdp.70002)
Supplement: Supplementary file 1 — Data S1 [file BJDP-44-33-s001.docx]

Content

[**S1 Documentation of film stimuli used in the study** 2](#_Toc193204506)

[**S2 Model equation** 11](#_Toc193204507)

[**S3.1 Predicting target-emotion intensity** 13](#_Toc193204508)

[Model specification and estimation 13](#_Toc193204509)

[Model selection 15](#_Toc193204510)

[Results table for emotional intensity 16](#_Toc193204511)

[**S3.2 Predicting emotional specificity** 28](#_Toc193204512)

[Model specification and estimation 28](#_Toc193204513)

[Model selection 31](#_Toc193204514)

[Results table for emotional specificity 33](#_Toc193204515)

[**S3.3 Follow-up analyses: Effects across individual film clips** 48](#_Toc193204516)

[Model specification and estimation 48](#_Toc193204517)

[Model selection 52](#_Toc193204518)

[Results tables for heterogeneity analyses 55](#_Toc193204519)

**S1 Documentation of film stimuli used in the study**

| Target Emotion |  | Film Clip | Description | Length in seconds | Source  (for movies, the start and end time of the clip is indicated in brackets) |
| --- | --- | --- | --- | --- | --- |
| Fear | * | Copycat | A woman enters a crime scene. One of the victims begins to move as she turns her back to him. | 57 | Amiel, J. (Director). (1995). *Copycat* [Film]. Regency Enterprises (01:44:17-01:45:14). |
|  |  |  |  |  |  |
| Fear | * | Silence of the Lambs | In total darkness, a woman is being followed by a person wearing a night observation device. | 101 | Demme, J. (Director). (1991). *The Silence of the Lambs* [Film]. Strong Heart Productions (01:47:27-01:49:08). |
|  |  |  |  |  |  |
| Fear | * | IT | A clown sitting in a gully hole tries to lure a little boy into following him. | 101 | Cohen, L. D. (Writer), King, S. (Writer), & Wallace, T. L. (Writer & Director). (1990, November 18). Part 1 [Miniseries episode]. In Basino, M., Epstein, A. S., & Green, J. (Executive Producers), *Stephen Kings It*. Lorimar Television; DawnField Enterntainment; TheKonigsberg; Sanitsky Company; Green/Epstein Productions (10:12-11:53). |
|  |  |  |  |  |  |
| Fear | * | Halloween | A woman is climbing a dark flight of stairs. Threatening music suggests something bad is about to happen. | 78 | Carpenter, J. (Director) (1978) *Halloween* [Film]. Compass International Pictures; Falcon International Productions (1:24:30-1:25:48). |
|  |  |  |  |  |  |
| Fear | * | Misery | A handcuffed man is about to be maltreated by a mad woman holding a sledge. | 114 | Reiner, R. (Director). (1990). *Misery* [Film]. Castle Rock Entertainment; Nelson Entertainment (1:17:34-1:19:28). |
|  |  |  |  |  |  |
| Fear | * | Rec | In panic, a woman is desperately trying to find her keys to get into a locked door. | 79 | Balagueró, J. & Plaza, P. (Directors). (2007). *Rec* [Film]. Casteleo (57:50-59:09). |
| *Note*. * stimulus was excluded for adolescents. *(*table continues on next page) | | | | | |
| Fear | * | Scream | A woman is hiding and running from a masked person in her house. | 96 | Craven, W. (Director) (1996). *Scream* [Film]. Woods Entertainment (08:55-10:31). |
|  |  |  |  |  |  |
| Fear | * | The Messengers | A woman is packing her bags in panic, telling her child to hide under the bed. Someone slowly turns the doorknob. | 36 | Pang, D. & Pang, O. (Directors). (2007) *The Messengers*. [Film]. Columbia Pictures; Screen Gems; Mandate Pictures; Ghost House Pictures (01:04-01:40). |
|  |  |  |  |  |  |
| Fear | * | The Shining (axe) | Mother and son try to escape from the father who pursues them with an axe. | 105 | Kubrick, S. (Director). (1980). *The* *Shining* [Film]. The Producer Circle Company; Peregrine Productions; Hawk Films (01:37:52-01:39:37). |
|  |  |  |  |  |  |
| Fear | * | The Shining (boy) | A little boy playing in a lonesome corridor approaches an unlocked door. | 82 | Kubrick, S. (Director). (1980). *The* *Shining* [Film]. The Producer Circle Company; Peregrine Productions; Hawk Films (42:47-44:09). |
| Fear | * | Room 1408 (skyscraper) | A man is losing his balance on the outer window sill high up on a skyscraper. | 28 | Håfström, M. (Director). (2007). 1408 [Film]. Dimension Films; Di Bonaventura Pictures (55:32-55:50). |
|  |  |  |  |  |  |
| Fear | * | Room 1408 (child’s voice) | A frightened man alone at home sees a scary-looking man appear in one corner. | 30 | Håfström, M. (Director). (2007). 1408 [Film]. Dimension Films; Di Bonaventura Pictures (58:40-59:10). |
|  |  |  |  |  |  |
| Happiness |  | Baby ponytail | A baby gets tickled by the mother's hair and laughs. | 10 | Public video platform |
| Happiness |  | Peekaboo | A mother plays "peekaboo" with her baby. | 39 | Public video platform |
| Happiness |  | Baby Tickle | A baby gets tickled by its father and laughs hysterically. | 15 | Public video platform |
| *Note*. * stimulus was excluded for adolescents. *(*table continues on next page) | | | | | |
| Happiness |  | Soccer | Soccer players celebrate after scoring at the soccer world championship. | 17 | Public video platform |
|  |  |  |  |  |  |
| Happiness |  | Proposal | A man proposes to his girlfriend by having a plane writing the proposal in the sky. | 49 | Public video platform |
|  |  |  |  |  |  |
| Happiness |  | Lemur | A lemur monkey raises his arms in pleasure as he is being caressed. | 24 | Public video platform |
| Happiness |  | Airport (grandparents) | A little boy runs to meet his grandparents at the airport. | 15 | Public video platform |
|  |  |  |  |  |  |
| Happiness |  | Airport (father) | Two boys run towards their father to hug him at the airport. | 18 | Public video platform |
|  |  |  |  |  |  |
| Anger | * | Shawcross | Interview with a murderer who shows no regret for having killed several children. | 57 | Public video platform |
|  |  |  |  |  |  |
| Anger | * | Subway beating | Two young men brutally beat a senior citizen in a subway station. | 55 | Public video platform |
|  |  |  |  |  |  |
| Anger |  | Cry Freedom | Armed forces prepare to shoot a peaceful demonstration. | 60 | Attenborough, R. (Director). (1978). Cry Freedom [Film]. Marble Arch Productions (02:20:40-02:21:40). |
|  |  |  |  |  |  |
| Anger | * | Funny Games | A family father man asks two teenage visitors to leave his house, but they refuse and start insulting him. | 43 | Haneke, M. (Director). (1997). *Funny Games*. [Film]. Oesterreichischer Rundfunk; Wega Film (23:06-23:49). |
| *Note*. * stimulus was excluded for adolescents. *(*table continues on next page) | | | | | |
| Anger | * | In my skin | A young woman is about to be tossed into a swimming pool by others. She is very upset and begs her friend for help, but the friend does not move. | 45 | de Van, M. (Director). (2002). *In my Skin*. [Film]. Lazennec 3; Canal + (32:30-33:15). |
|  |  |  |  |  |  |
| Anger |  | Mass panic | Two people at a German music festival show a lack of sympathy when they learn that many people have just died in a nearby mass panic. | 31 | Public video platform |
| Anger |  | Sarrazin | A German politician makes a racist statement in public. | 39 | Public video platform |
| Anger |  | Taxes | TV documentary on rich German people who evade taxes. | 80 | Public video platform |
|  |  |  |  |  |  |
| Anger |  | Police violence | TV documentary showing a violent police operation against an environmentalist demonstration. | 47 | Public video platform |
|  |  |  |  |  |  |
| Anger |  | BP CEO | After the Deepwater Horizon oil spill, the head of the BP company gives evasive answers when being questioned by the US congress. | 115 | Public video platform |
|  |  |  |  |  |  |
| Sadness |  | Armageddon goodbye | A father who is about to die says goodbye to his daughter over video call. | 66 | Bay, M. (Director). (1998). *Armageddon* [Film]. Touchstone Pictures; Jerry Bruckheimer Films (02:08:19-02:09:25). |
|  |  |  |  |  |  |
| Sadness |  | Bambi | Deer Bambi's mother is shot dead by hunters. | 147 | Hand, D. (Director). (1942). *Bambi*. Walt Disney Animation Studios (40:30-42:11, 42:31-43:17). |
| *Note*. * stimulus was excluded for adolescents. *(*table continues on next page) | | | | | |
| Sadness |  | Hospital | A woman starts crying at the hospital bed of her child who is terminally ill. | 53 | Cassavetes, N. (Director). (2009). *My sister's keeper* [Film]. Gran Via Productions; Relativity Media; Imagine Entertainment; The Kennedy/ Marshall Company; Mark Johnson Production; Curmudgeon Films (01:31:44-01:32:37). |
|  |  |  |  |  |  |
| Sadness | * | Cancer diagnosis | A couple learns from their doctor that the man has cancer. | 36 | Gilligan, V. (Writer), & McKay, J. (Director). (2008, February 17). Cancer man [Die Diagnose] (Season 1, Episode 4) [TV series episode]. In V. Gilligan & M. Johnson (Executive Producers), *Breaking Bad*. High Bridge Productions; Gran Via Productions; Sony Pictures Television (36:59-37:35). |
|  |  |  |  |  |  |
| Sadness | * | Cancer therapy | A man with cancer tells his family that he rejects further medical treatment. | 76 | Lin, P. (Writer), & Brock, T. (Director). (2008, February 24). Gray matter [Grauzonen] (Season 1, Episode 5) [TV series episode]. In V. Gilligan & M. Johnson (Executive Producers), *Breaking Bad*. High Bridge Productions; Gran Via Productions; Sony Pictures Television (35:30-36:46). |
|  |  |  |  |  |  |
| Sadness |  | Death of a classmate | Students in a school class are told that one of their classmates has died. | 45 | Smith, J. N. (Director). (1995). *Dangerous Minds* [Film]. Hollywood Pictures; Jerry Bruckheimer Films (01:20:16-01:21:01). |
|  |  |  |  |  |  |
| Sadness |  | Lion King | A young lion tries to make his dead father get up and starts to cry. | 87 | Allers, R. & Minkoff, R. (Director). (1994). *The Lion King* [Film]. Walt Disney Studios Motion Pictures USA; Fairview Entertainment (35:23-36:50). |
|  |  |  |  |  |  |
| Sadness |  | Sam | A father is desperately hugging his daughter as the authorities start to separate them. | 62 | Nelson, J. (Director). (2001). *I am Sam* [Film]. New Line Cinema (01:30:22-01:31:24). |
| *Note*. * stimulus was excluded for adolescents. *(*table continues on next page) | | | | | |
| Sadness | * | Car accident | A young boy is killed by a car. His father rushes to the scene and mourns his son. | 54 | DUI intervention video, public video platform (00:00-00:54) |
|  |  |  |  |  |  |
| Sadness |  | Radio | A mentally disabled young man hears about his mother's death and cries over her photograph. | 96 | Tollin, M. (Director). (2003). *Radio* [Film]. Tollin/ Robbins Prod; Revolution Studios (01:14:12-01:14:21, 01:16:32- 01:17:59). |
|  |  |  |  |  |  |
| Sadness |  | City of angels | A woman dies in the arms of a man after they have told each other that they were the best that ever happened to them. | 142 | Silberling, B. (Director). (1998). *City of angels* [Film]. Atlas Entertainment; Monarchy Enterprises B.V.; Regency Enterprises; Taurus Films; Warner Bros (40:20-42:42). |
|  |  |  |  |  |  |
| Sadness |  | Champ | A son watches his mentor's death after a boxing match. | 130 | Zeffirelli, F. (Director). (1979). *The Champ* [Film]. Metro Goldwyn Mayer (48:01-48:23, 49:25- 49:52, 51:02 - 52:23). |
|  |  |  |  |  |  |
| Sadness | * | Dead poets | Two parents find their son who has committed suicide. | 137 | Weir, P. (Director). (1989). *Dead poets society* [Film]. Silver Screen Partners IV; Touchstone Pictures (45:00-47:17). |
|  |  |  |  |  |  |
| Sadness |  | Return to me | After an accident, a man rushes his wife to the hospital. Back home, he watches his dog wait for his wife. He cries as he tells the dog that the wife will never come home again | 181 | Hunt, B. (Director). (2000). *Return to me* [Film]. Metro Goldwyn Mayer (13:20-14:05, 17:20-19:36). |
|  |  |  |  |  |  |
| Disgust |  | Nose picking politician | A politician is picking his nose. | 14 | Public video platform |
| *Note*. * stimulus was excluded for adolescents. *(*table continues on next page) | | | | | |
|  | | | | | |
| Disgust |  | Sneeze | A man sneezes strongly in his hands and looks at them with disgust. | 20 | Public video platform |
|  |  |  |  |  |  |
| Disgust |  | Butter | A man is eating a whole package of pure butter. | 25 | Public video platform |
|  |  |  |  |  |  |
| Disgust | * | Indiana Jones | A man and a woman are entering a cave full of rats. | 82 | Spielberg, S. (Director). (1989). *Indiana Jones and the last crusade* [Film]. Lucasfilm Ltd.; Paramount Pictures (32:33-33:55). |
|  |  |  |  |  |  |
| Disgust |  | Nose picking soccer coach | A soccer coach is picking his nose. | 18 | Public video platform |
|  |  |  |  |  |  |
| Disgust |  | Maggots | Maggots moving in a box. | 38 | Own recording |
|  |  |  |  |  |  |
| Disgust |  | Pimple | A young man is squeezing a huge pimple in his face. | 17 | Own recording |
|  |  |  |  |  |  |
| Disgust | * | Octopus | A man is eating a live octopus. | 16 | Chan-wook, P. (Director). (2003). *Oldboy* [Film]. Wild Side (26:40-26:56). |
|  |  |  |  |  |  |
| Disgust |  | Ribs | The ribs of a very thin man are protruding as he is breathing in and out. | 39 | Public video platform |
|  |  |  |  |  |  |
| Disgust |  | Snot | A young boy is playing with his snot. | 14 | Public video platform |
| *Note*. * stimulus was excluded for adolescents. *(*table continues on next page) | | | | | |
| Disgust | * | Trainspotting | A man dives into a filthy toilet. | 46 | Boyle, D. (Director). (1996). *Trainspotting* [Film]. Channel Four Films; PolyGram Filmed Entertainment; Figment Films (08:16-09:02). |
|  |  |  |  |  |  |
| Neutral |  | Bush | Close-up view of a bush. | 35 | Own recording |
|  |  |  |  |  |  |
| Neutral |  | Blue (desk) | A man clears out the drawers of his desk. A woman is walking down a street. | 40 | Kieślowski, K. (Director). (1993). *Trois couleurs: Bleu* [Film]. CAB Productions; CED Productions; France 3 Cinéma; MK2 Productions; Tor Productions; Eurimages (Fonds du Conseil de l’Europe, 12:13-12:53). |
|  |  |  |  |  |  |
| Neutral |  | Blue (escalator) | A woman carrying a box uses an escalator. | 25 | Kieślowski, K. (Director). (1993). *Trois couleurs: Bleu* [Film]. CAB Productions; CED Productions; France 3 Cinéma; MK2 Productions; Tor Productions; Eurimages (Fonds du Conseil de l’Europe, 27:35-28:00). |
|  |  |  |  |  |  |
| Neutral |  | Screensaver | Screensaver with changing color bars. | 55 | Gross & Levenson (1995). *Color Bars* [Video]. Stanford. https://stanford.app.box.com/s/ry3ywherpxaflov6i94ohum8xnnce7gi |
|  |  |  |  |  |  |
| Neutral |  | Moving lines | Various lines in different colors appear and disappear. | 43 | Gross & Levenson (1995). *Abstract Shapes* [Video]. Stanford. <https://stanford.app.box.com/s/ujsdw836qshfe9cyhu89e0otvga539en> (00:02-00:45). |
|  |  |  |  |  |  |
| Neutral |  | Crossroads | Cars passing a crossroads. | 55 | Own recording |
| *Note*. * stimulus was excluded for adolescents. *(*table continues on next page) | | | | | |
|  |  |  |  |  |  |
| Neutral |  | Duck pond | A view of a lake with ducks. | 53 | Own recording |
|  |  |  |  |  |  |
|  |  |  |  |  |  |
| Neutral |  | Car ride | A woman rides in a car, gets off and knocks on a door. A man opens and lets her in. | 43 | Annaud, J. (Director). (1992). *L’Amant* [Film]. Renn Productions; Films A2; Timothy Burrill Productions Limited; Grai Phang Film Studio, 51:34-52:17). |
|  |  |  |  |  |  |
| Neutral |  | Potato harvest | An agricultural machine is harvesting potatoes. | 61 | Public video platform |

**S2 Model equation**

The model for predicting target-emotion intensity (H1 and H2) is represented by the following equations, exemplarily for older age group and neutral condition as reference. An analogue model was used to predict emotion specificity (H3).

$$y_{ijk}=\beta_{0jk}+\beta_{1}\left( Agegroup_{MiddleAged} \right)+\beta_{2}\left( Agegroup_{Younger} \right)+\beta_{3}\left( Agegroup_{Adolescent} \right)+\beta_{4j}\left( Emotion_{Anger} \right)+\beta_{5j}\left( Emotion_{Disgust} \right)+\beta_{6j}\left( Emotion_{Fear} \right)+\beta_{7j}\left( Emotion_{Happiness} \right)+\beta_{8j}\left( Emotion_{Sadness} \right)+\beta_{9}\left( Agegroup_{MiddleAged}\times Emotion_{Anger} \right)+\beta_{10}\left( Agegroup_{Younger}\times Emotion_{Anger} \right)+\beta_{11}\left( Agegroup_{Adolescent}\times Emotion_{Anger} \right)+\beta_{12}\left( Agegroup_{MiddleAged}\times Emotion_{Disgust} \right)+\beta_{13}\left( Agegroup_{Younger}\times Emotion_{Disgust} \right)+\beta_{14}\left( Agegroup_{Adolescent}\times Emotion_{Disgust} \right)+\beta_{15}\left( Agegroup_{MiddleAged}\times Emotion_{Fear} \right)+\beta_{16}\left( Agegroup_{Younger}\times Emotion_{Fear} \right)+\beta_{17}\left( Agegroup_{Adolescent}\times Emotion_{Fear} \right)+\beta_{18}\left( Agegroup_{MiddleAged}\times Emotion_{Happiness} \right)+\beta_{19}\left( Agegroup_{Younger}\times Emotion_{Happiness} \right)+\beta_{20}\left( Agegroup_{Adolescent}\times Emotion_{Happiness} \right)+\beta_{21}\left( Agegroup_{MiddleAged}\times Emotion_{Sadness} \right)+\beta_{22}\left( Agegroup_{Younger}\times Emotion_{Sadness} \right)+\beta_{23}\left( Agegroup_{Adolescent}\times Emotion_{Sadness} \right)+\varepsilon_{\mathrm{ijk}}$$

$$\beta_{0jk}=\gamma_{0}+u_{0j}+v_{0k}$$

$$\beta_{4j}=\gamma_{4}+u_{4j}$$

$$\beta_{5j}=\gamma_{5}+u_{5j}$$

$$\beta_{6j}=\gamma_{6}+u_{6j}$$

$$\beta_{7j}=\gamma_{7}+u_{7j}$$

$$\beta_{8j}=\gamma_{8}+u_{8j}$$

$$u_{j}=\left( u_{0j},u_{4j},u_{5j},u_{6j},u_{7j},u_{8j} \right)\mathcal{\sim N}\left( 0,D \right)$$

$$v_{0k}\mathcal{\sim N}\left( 0,\sigma_{v_{0k}}^{2} \right)$$

$$\varepsilon_{\mathrm{ijk}}\sim\mathcal{N}\left( 0,\sigma_{\varepsilon_{ijk}}^{2} \right)$$

Where $y_{ijk}$ is the target-emotion intensity rating (resp. emotion specificity) of the targeted emotion at occasion *i* from subject *j* of video *k*, $\beta_{0jk}$to$\beta_{8jk}$are the random coefficients, $\beta_{9}$ to $\beta_{23}$ are the fixed interaction terms and $\varepsilon_{ijk}$is the random residual term. The variable $\gamma_{m}$ represents the fixed part of the *m*^th^ random coefficient ($\beta_{0jk}$to$\beta_{8jk}$), where $\gamma_{0}$ is the fixed intercept (i.e., the expected mean rating of an older adult of a video of the neutral condition). The variable $u_{mj}$represents the random residual term of the *m* ^th^ random coefficient ($\beta_{0jk}$to$\beta_{8jk}$) for subject *j*; $v_{0k}$ is the random residual term for video *k*. All random effects were assumed to follow a normal distribution (denoted by $\mathcal{\sim N}\left( \mu,\sigma^{2} \right)$). $D$ represents the variance-covariance matrix for the set of random effects $u_{j}$.

**S3 R Code and Results Tables**

## Install and attach necessary packages ##

packages = c("tidyverse","haven","lmerTest","magrittr","lmtest","knitr","kableExtra","sjPlot")

# "janitor","rlist","XML","knitr","lattice","rcompanion","cAIC4","interactions","plyr","dotwhisker","cowplot")

install.packages(setdiff(packages,rownames(installed.packages())))

lapply(packages,require, character.only=T, quietly = T)

D_FE1 <- read_sav("videodata_complete.sav")

### Data wrangling ###

## converting ID, agegroup, emotion and video into factors ##

D_FE1$ID %<>% factor()

D_FE1$agegroup %<>%

factor(labels = c("teen","young_ad","middle_ad","old_ad")) #%>%

D_FE1$emotion %<>%

factor(labels = c("sad","angry","disgusted","afraid","joyful","neutral")) %>%

relevel(ref = "neutral")

D_FE1$video %<>%

factor()

D_FE1 %<>%

dplyr::rename("sad" = "sadness",

"joyful" = "happiness",

"disgusted" = "disgust",

"afraid" = "fear",

"angry" = "anger",

"amused" = "amusement",

"surprised" = "surprise",

"shamed" = "shame",

"neutral" = "neutral")

**S3.1 Predicting target-emotion intensity**

## Model specification and estimation

The dependent variable is the rating on the target emotion, named targetemorating.
The model is repeated four times, with neutral as reference category throughout all models. However, the dummy codes for age group are recoded, making each age group the reference group in one of the models.

The intercept represents the mean target-emotion rating for *neutral* film clips for the age group that serves as reference group. The main effects for the *age contrasts* are the difference between the mean of the contrasted group and the reference group (*contrast group - reference group*) on the *neutral* rating. The main effect of the *emotion* is the difference between the mean of the respective emotion and the mean of the neutral condition, both across all age groups. The interaction terms indicate that the rating for the respective emotion differ in the respective age group, compared to the rating of the *neutral* condition.

### Model Specification - targetemorating ###

## Specification of all models in ascending order of complexity ##

M0.Targ = 'targetemorating ~ 1 + (1|ID) + (1|video)'

M1.Targ = 'targetemorating ~ agegroup+emotion + (1|ID) + (1|video)'

M2.Targ = 'targetemorating ~ agegroup+emotion+agegroup:emotion + (1|ID) + (1|video)'

M3.Targ = 'targetemorating ~ agegroup+emotion+agegroup:emotion + (1+emotion|ID) + (1|video)'

M4.Targ = 'targetemorating ~ agegroup+emotion+agegroup:emotion + (1+emotion|ID) + (1+agegroup|video)'

## set contrast 1 - Older. vs all ##

D_FE1$agegroup %<>% factor(levels=c("old_ad","middle_ad","young_ad","teen"))

contrasts(D_FE1$agegroup) = matrix(c(0,1,0,0,

0,0,1,0,

0,0,0,1),nrow=4,ncol=3)

dimnames(contrasts(D_FE1$agegroup))[[2]] = c("Older_vs_middle","Older_vs_Younger","Older_vs_teen")

Fit0.Targ.Older = lmer(M0.Targ,D_FE1,REML=TRUE,na.action="na.exclude")

Fit1.Targ.Older = lmer(M1.Targ,D_FE1,REML = TRUE,na.action = "na.exclude")

Fit2.Targ.Older = lmer(M2.Targ,D_FE1,REML = TRUE,na.action = "na.exclude")

Fit3.Targ.Older = lmer(M3.Targ,D_FE1,REML = TRUE,na.action = "na.exclude")

Fit4.Targ.Older = lmer(M4.Targ,D_FE1,REML = TRUE,na.action = "na.exclude")

## set contrast 2 - middle. vs all ##

D_FE1$agegroup %<>% factor(levels=c("middle_ad","old_ad","young_ad","teen"))

contrasts(D_FE1$agegroup) = matrix(c(0,1,0,0,

0,0,1,0,

0,0,0,1),nrow=4,ncol=3)

dimnames(contrasts(D_FE1$agegroup))[[2]] = c("middle_vs_Older","middle_vs_Younger","middle_vs_teen")

Fit0.Targ.middle = lmer(M0.Targ,D_FE1,REML=TRUE,na.action="na.exclude")

Fit1.Targ.middle = lmer(M1.Targ,D_FE1,REML = TRUE,na.action = "na.exclude")

Fit2.Targ.middle = lmer(M2.Targ,D_FE1,REML = TRUE,na.action = "na.exclude")

Fit3.Targ.middle = lmer(M3.Targ,D_FE1,REML = TRUE,na.action = "na.exclude")

Fit4.Targ.middle = lmer(M4.Targ,D_FE1,REML = TRUE,na.action = "na.exclude")

## set contrast 3 - Younger. vs all ##

D_FE1$agegroup %<>% factor(levels=c("young_ad","old_ad","middle_ad","teen"))

contrasts(D_FE1$agegroup) = matrix(c(0,1,0,0,

0,0,1,0,

0,0,0,1),nrow=4,ncol=3)

dimnames(contrasts(D_FE1$agegroup))[[2]] = c("Younger_vs_Older","Younger_vs_middle","Younger_vs_teen")

Fit0.Targ.Younger = lmer(M0.Targ,D_FE1,REML=TRUE,na.action="na.exclude")

Fit1.Targ.Younger = lmer(M1.Targ,D_FE1,REML = TRUE,na.action = "na.exclude")

Fit2.Targ.Younger = lmer(M2.Targ,D_FE1,REML = TRUE,na.action = "na.exclude")

Fit3.Targ.Younger = lmer(M3.Targ,D_FE1,REML = TRUE,na.action = "na.exclude")

Fit4.Targ.Younger = lmer(M4.Targ,D_FE1,REML = TRUE,na.action = "na.exclude")

## set contrast 4 - teen vs all ##

D_FE1$agegroup %<>% factor(levels=c("teen","old_ad","middle_ad","young_ad"))

contrasts(D_FE1$agegroup) = matrix(c(0,1,0,0,

0,0,1,0,

0,0,0,1),nrow=4,ncol=3)

dimnames(contrasts(D_FE1$agegroup))[[2]] = c("teen_vs_Older","teen_vs_middle","teen_vs_Younger")

Fit0.Targ.teen = lmer(M0.Targ,D_FE1,REML=TRUE,na.action="na.exclude")

Fit1.Targ.teen = lmer(M1.Targ,D_FE1,REML = TRUE,na.action = "na.exclude")

Fit2.Targ.teen = lmer(M2.Targ,D_FE1,REML = TRUE,na.action = "na.exclude")

Fit3.Targ.teen = lmer(M3.Targ,D_FE1,REML = TRUE,na.action = "na.exclude")

Fit4.Targ.teen = lmer(M4.Targ,D_FE1,REML = TRUE,na.action = "na.exclude")

## Model selection

Checking for singularity of models. Singularity indicates problems with parameter estimation, often arising from high collinearity or correlation among the predictors. To address this issue, we made the decision to exclude models that consistently yielded singularity across all defined contrasts. However, we adopted a slightly different approach when only one specific defined contrast led to singularity. In such cases, we exercised caution but decided to retain the model, considering the possibility that singularity may be driven by unique characteristics or relationships captured by that contrast. In the case of a retained model with a singular fit, this is additionally mentioned after the result tables. For the remaining models, likelihood-ratio-test indicate which terms are meaningful.

### check singularity of models ###

Fit4.Targ.Older %>% isSingular()

## [1] FALSE

Fit4.Targ.middle %>% isSingular()

## [1] FALSE

Fit4.Targ.Younger %>% isSingular()

## [1] TRUE

Fit4.Targ.teen %>% isSingular()

## [1] FALSE

# Model 4 is not singular, but has convergence problems

Fit3.Targ.Older %>% isSingular()

## [1] FALSE

Fit3.Targ.middle %>% isSingular()

## [1] FALSE

Fit3.Targ.Younger %>% isSingular()

## [1] FALSE

Fit3.Targ.teen %>% isSingular()

## [1] FALSE

### check model improvement ###

lrtest(Fit3.Targ.Older,

Fit2.Targ.Older,

Fit1.Targ.Older,

Fit0.Targ.Older)

## Likelihood ratio test

##

## Model 1: targetemorating ~ agegroup + emotion + agegroup:emotion + (1 +

## emotion | ID) + (1 | video)

## Model 2: targetemorating ~ agegroup + emotion + agegroup:emotion + (1 |

## ID) + (1 | video)

## Model 3: targetemorating ~ agegroup + emotion + (1 | ID) + (1 | video)

## Model 4: targetemorating ~ 1 + (1 | ID) + (1 | video)

## #Df LogLik Df Chisq Pr(>Chisq)

## 1 46 -11294

## 2 26 -11768 -20 948.500 < 2e-16 ***

## 3 12 -11842 -14 147.999 < 2e-16 ***

## 4 4 -11851 -8 18.171 0.01998 *

## ---

## Signif. codes: 0 '***' 0.001 '**' 0.01 '*' 0.05 '.' 0.1 ' ' 1

# test significance of random effects

ranova(Fit3.Targ.Older)

## ANOVA-like table for random-effects: Single term deletions

##

## Model:

## targetemorating ~ agegroup + emotion + (1 + emotion | ID) + (1 | video) + agegroup:emotion

## npar logLik AIC LRT Df Pr(>Chisq)

## <none> 46 -11294 22679

## emotion in (1 + emotion | ID) 26 -11768 23588 948.50 20 < 2.2e-16 ***

## (1 | video) 45 -11580 23250 573.06 1 < 2.2e-16 ***

## ---

## Signif. codes: 0 '***' 0.001 '**' 0.01 '*' 0.05 '.' 0.1 ' ' 1

## Results table for emotional intensity

## Creating output table. Note that adolescents did not watch any fear films. ##

tab_model(Fit3.Targ.Older, Fit3.Targ.middle, Fit3.Targ.Younger,Fit3.Targ.teen, show.re.var = T,show.icc = F,

dv.labels = c('Reference: Older','Reference: Middle-aged','Reference: Younger','Reference: Adolescent'))

|  | **Reference: Older** | | | **Reference: Middle-aged** | | | **Reference: Younger** | | | **Reference: Adolescents** | | |
| --- | --- | --- | --- | --- | --- | --- | --- | --- | --- | --- | --- | --- |
| *Predictors* | *Estimates* | *CI* | *p* | *Estimates* | *CI* | *p* | *Estimates* | *CI* | *p* | *Estimates* | *CI* | *p* |
| (Intercept) | 3.56 | 2.87 – 4.24 | **<0.001** | 4.01 | 3.28 – 4.74 | **<0.001** | 4.19 | 3.51 – 4.87 | **<0.001** | 4.24 | 3.58 – 4.90 | **<0.001** |
| Age group: Older vs middle | 0.45 | -0.39 – 1.29 | 0.291 |  |  |  |  |  |  |  |  |  |
| Age group: Older vs younger | 0.63 | -0.17 – 1.44 | 0.123 |  |  |  |  |  |  |  |  |  |
| Age group: Older vs teen | 0.68 | -0.10 – 1.47 | 0.087 |  |  |  |  |  |  |  |  |  |
| emotion [sad] | 1.29 | 0.42 – 2.16 | **0.004** | 0.55 | -0.37 – 1.48 | 0.243 | -0.36 | -1.23 – 0.52 | 0.422 | -0.09 | -0.94 – 0.76 | 0.833 |
| emotion [angry] | 0.38 | -0.53 – 1.29 | 0.418 | -0.08 | -1.05 – 0.88 | 0.869 | -1.00 | -1.91 – -0.09 | **0.031** | -1.50 | -2.39 – -0.61 | **0.001** |
| emotion [disgusted] | 0.39 | -0.53 – 1.32 | 0.405 | -0.16 | -1.15 – 0.83 | 0.749 | -0.36 | -1.29 – 0.57 | 0.446 | -0.04 | -0.94 – 0.86 | 0.931 |
| emotion [afraid] | 0.13 | -0.85 – 1.11 | 0.794 | -0.26 | -1.30 – 0.79 | 0.633 | -1.47 | -2.45 – -0.48 | **0.004** | -1.47 | -2.45 – -0.48 | **0.004** |
| emotion [joyful] | 0.79 | -0.15 – 1.73 | 0.098 | -0.27 | -1.27 – 0.73 | 0.597 | -1.16 | -2.10 – -0.21 | **0.016** | -0.82 | -1.73 – 0.08 | 0.075 |
| Age group: Older vs middle × emotion [sad] | -0.74 | -1.80 – 0.33 | 0.174 |  |  |  |  |  |  |  |  |  |
| Age group: Older vs Younger × emotion [sad] | -1.65 | -2.66 – -0.63 | **0.002** |  |  |  |  |  |  |  |  |  |
| Age group: Older vs teen × emotion [sad] | -1.38 | -2.37 – -0.39 | **0.007** |  |  |  |  |  |  |  |  |  |
| Age group: Older vs middle × emotion [angry] | -0.46 | -1.55 – 0.63 | 0.412 |  |  |  |  |  |  |  |  |  |
| Age group: Older vs younger × emotion [angry] | -1.38 | -2.42 – -0.33 | **0.010** |  |  |  |  |  |  |  |  |  |
| Age group: Older vs teen × emotion [angry] | -1.88 | -2.90 – -0.85 | **<0.001** |  |  |  |  |  |  |  |  |  |
| Age group: Older vs middle × emotion [disgusted] | -0.56 | -1.69 – 0.58 | 0.338 |  |  |  |  |  |  |  |  |  |
| Age group: Older vs younger × emotion [disgusted] | -0.76 | -1.84 – 0.33 | 0.173 |  |  |  |  |  |  |  |  |  |
| Age group: Older vs teen × emotion [disgusted] | -0.43 | -1.50 – 0.63 | 0.424 |  |  |  |  |  |  |  |  |  |
| Age group: Older vs middle × emotion [afraid] | -0.39 | -1.63 – 0.86 | 0.542 |  |  |  |  |  |  |  |  |  |
| Age group: Older vs younger × emotion [afraid] | -1.60 | -2.78 – -0.41 | **0.009** |  |  |  |  |  |  |  |  |  |
| Age group: Older vs middle × emotion [joyful] | -1.06 | -2.19 – 0.07 | 0.066 |  |  |  |  |  |  |  |  |  |
| Age group: Older vs Younger × emotion [joyful] | -1.95 | -3.03 – -0.87 | **<0.001** |  |  |  |  |  |  |  |  |  |
| Age group: Older vs teen × emotion [joyful] | -1.62 | -2.67 – -0.56 | **0.003** |  |  |  |  |  |  |  |  |  |
| Age group: middle vs older |  |  |  | -0.45 | -1.29 – 0.39 | 0.291 |  |  |  |  |  |  |
| Age group: middle vs younger |  |  |  | 0.18 | -0.66 – 1.02 | 0.675 |  |  |  |  |  |  |
| Age group: middle vs teen |  |  |  | 0.23 | -0.59 – 1.05 | 0.583 |  |  |  |  |  |  |
| Age group: middle vs older × emotion [sad] |  |  |  | 0.74 | -0.33 – 1.80 | 0.174 |  |  |  |  |  |  |
| Age group: middle vs younger × emotion [sad] |  |  |  | -0.91 | -1.97 – 0.16 | 0.094 |  |  |  |  |  |  |
| Age group: middle vs teen × emotion [sad] |  |  |  | -0.64 | -1.69 – 0.40 | 0.227 |  |  |  |  |  |  |
| Age group: middle vs older × emotion [angry] |  |  |  | 0.46 | -0.63 – 1.55 | 0.412 |  |  |  |  |  |  |
| Age group: middle vs younger × emotion [angry] |  |  |  | -0.92 | -2.01 – 0.17 | 0.099 |  |  |  |  |  |  |
| Age group: middle vs teen × emotion [angry] |  |  |  | -1.42 | -2.50 – -0.34 | **0.010** |  |  |  |  |  |  |
| Age group: middle vs older × emotion [disgusted] |  |  |  | 0.56 | -0.58 – 1.69 | 0.338 |  |  |  |  |  |  |
| Age group: middle vs Younger × emotion [disgusted] |  |  |  | -0.20 | -1.34 – 0.94 | 0.730 |  |  |  |  |  |  |
| Age group: middle vs teen × emotion [disgusted] |  |  |  | 0.12 | -0.99 – 1.24 | 0.831 |  |  |  |  |  |  |
| Age group: middle vs older × emotion [afraid] |  |  |  | 0.39 | -0.86 – 1.63 | 0.542 |  |  |  |  |  |  |
| Age group: middle vs younger × emotion [afraid] |  |  |  | -1.21 | -2.45 – 0.03 | 0.057 |  |  |  |  |  |  |
| gegroupmiddle vs older × emotion [joyful] |  |  |  | 1.06 | -0.07 – 2.19 | 0.066 |  |  |  |  |  |  |
| Age group: middle vs younger × emotion [joyful] |  |  |  | -0.89 | -2.02 – 0.24 | 0.124 |  |  |  |  |  |  |
| Age group: middle vs teen × emotion [joyful] |  |  |  | -0.55 | -1.66 – 0.55 | 0.325 |  |  |  |  |  |  |
| Age group: Younger vs older |  |  |  |  |  |  | -0.63 | -1.44 – 0.17 | 0.123 |  |  |  |
| Age group: Younger vs middle |  |  |  |  |  |  | -0.18 | -1.02 – 0.66 | 0.675 |  |  |  |
| Age group: Younger vs teen |  |  |  |  |  |  | 0.05 | -0.73 – 0.83 | 0.901 |  |  |  |
| Age group: Younger vs older × emotion [sad] |  |  |  |  |  |  | 1.65 | 0.63 – 2.66 | **0.002** |  |  |  |
| Age group: Younger vs middle × emotion [sad] |  |  |  |  |  |  | 0.91 | -0.16 – 1.97 | 0.094 |  |  |  |
| Age group: Younger vs teen × emotion [sad] |  |  |  |  |  |  | 0.27 | -0.73 – 1.26 | 0.600 |  |  |  |
| Age group: Younger vs older × emotion [angry] |  |  |  |  |  |  | 1.38 | 0.33 – 2.42 | **0.010** |  |  |  |
| Age group: Younger vs middle × emotion [angry] |  |  |  |  |  |  | 0.92 | -0.17 – 2.01 | 0.099 |  |  |  |
| Age group: Younger vs teen × emotion [angry] |  |  |  |  |  |  | -0.50 | -1.53 – 0.53 | 0.343 |  |  |  |
| Age group: Younger vs older × emotion [disgusted] |  |  |  |  |  |  | 0.76 | -0.33 – 1.84 | 0.173 |  |  |  |
| Age group: Younger vs middle × emotion [disgusted] |  |  |  |  |  |  | 0.20 | -0.94 – 1.34 | 0.730 |  |  |  |
| Age group: Younger vs teen × emotion [disgusted] |  |  |  |  |  |  | 0.32 | -0.74 – 1.39 | 0.554 |  |  |  |
| Age group: Younger vs older × emotion [afraid] |  |  |  |  |  |  | 1.60 | 0.41 – 2.78 | **0.009** |  |  |  |
| Age group: Younger vs middle × emotion [afraid] |  |  |  |  |  |  | 1.21 | -0.03 – 2.45 | 0.057 |  |  |  |
| Age group: Younger vs older × emotion [joyful] |  |  |  |  |  |  | 1.95 | 0.87 – 3.03 | **<0.001** |  |  |  |
| Age group: Younger vs middle × emotion [joyful] |  |  |  |  |  |  | 0.89 | -0.24 – 2.02 | 0.125 |  |  |  |
| Age group: Younger vs teen × emotion [joyful] |  |  |  |  |  |  | 0.33 | -0.72 – 1.39 | 0.535 |  |  |  |
| Age group: teen vs older |  |  |  |  |  |  |  |  |  | -0.68 | -1.47 – 0.10 | 0.087 |
| Age group: teen vs middle |  |  |  |  |  |  |  |  |  | -0.23 | -1.05 – 0.59 | 0.583 |
| Age group: teen vs Younger |  |  |  |  |  |  |  |  |  | -0.05 | -0.83 – 0.73 | 0.901 |
| Age group: teen vs Older × emotion [sad] |  |  |  |  |  |  |  |  |  | 1.38 | 0.39 – 2.38 | **0.007** |
| Age group: teen vs middle × emotion [sad] |  |  |  |  |  |  |  |  |  | 0.64 | -0.40 – 1.69 | 0.227 |
| Age group: teen vs younger × emotion [sad] |  |  |  |  |  |  |  |  |  | -0.27 | -1.26 – 0.73 | 0.600 |
| Age group: teen vs older × emotion [angry] |  |  |  |  |  |  |  |  |  | 1.88 | 0.85 – 2.90 | **<0.001** |
| Age group: teen vs middle × emotion [angry] |  |  |  |  |  |  |  |  |  | 1.42 | 0.34 – 2.50 | **0.010** |
| Age group: teen vs younger × emotion [angry] |  |  |  |  |  |  |  |  |  | 0.50 | -0.53 – 1.53 | 0.343 |
| Age group: teen vs older × emotion [disgusted] |  |  |  |  |  |  |  |  |  | 0.43 | -0.63 – 1.50 | 0.424 |
| Age group: teen vs middle × emotion [disgusted] |  |  |  |  |  |  |  |  |  | -0.12 | -1.24 – 0.99 | 0.831 |
| Age group: teen vs younger × emotion [disgusted] |  |  |  |  |  |  |  |  |  | -0.32 | -1.39 – 0.74 | 0.554 |
| Age group: teen vs older × emotion [afraid] |  |  |  |  |  |  |  |  |  | 1.60 | 0.41 – 2.79 | **0.009** |
| Age group: teen vs middle × emotion [afraid] |  |  |  |  |  |  |  |  |  | 1.21 | -0.03 – 2.45 | 0.057 |
| Age group: teen vs older × emotion [joyful] |  |  |  |  |  |  |  |  |  | 1.62 | 0.56 – 2.67 | **0.003** |
| Age group: teen vs middle × emotion [joyful] |  |  |  |  |  |  |  |  |  | 0.55 | -0.55 – 1.66 | 0.325 |
| Age group: teen vs younger × emotion [joyful] |  |  |  |  |  |  |  |  |  | -0.33 | -1.39 – 0.72 | 0.535 |
| **Random Effects** | | | | | | | | | | | | |
| σ^2^ | 2.33 | | | 2.33 | | | 2.33 | | | 2.33 | | |
| τ_00_ | 1.87 _ID_ | | | 1.87 _ID_ | | | 1.87 _ID_ | | | 1.87 _ID_ | | |
|  | 0.37 _video_ | | | 0.37 _video_ | | | 0.37 _video_ | | | 0.37 _video_ | | |
| τ_11_ | 2.96 _ID.emotionsad_ | | | 2.96 _ID.emotionsad_ | | | 2.96 _ID.emotionsad_ | | | 2.96 _ID.emotionsad_ | | |
|  | 3.07 _ID.emotionangry_ | | | 3.07 _ID.emotionangry_ | | | 3.07 _ID.emotionangry_ | | | 3.07 _ID.emotionangry_ | | |
|  | 3.39 _ID.emotiondisgusted_ | | | 3.39 _ID.emotiondisgusted_ | | | 3.39 _ID.emotiondisgusted_ | | | 3.39 _ID.emotiondisgusted_ | | |
|  | 4.16 _ID.emotionafraid_ | | | 4.16 _ID.emotionafraid_ | | | 4.16 _ID.emotionafraid_ | | | 4.16 _ID.emotionafraid_ | | |
|  | 3.30 _ID.emotionjoyful_ | | | 3.30 _ID.emotionjoyful_ | | | 3.30 _ID.emotionjoyful_ | | | 3.30 _ID.emotionjoyful_ | | |
| _01_ | -0.81 _ID.emotionsad_ | | | -0.81 _ID.emotionsad_ | | | -0.81 _ID.emotionsad_ | | | -0.81 _ID.emotionsad_ | | |
|  | -0.74 _ID.emotionangry_ | | | -0.74 _ID.emotionangry_ | | | -0.74 _ID.emotionangry_ | | | -0.74 _ID.emotionangry_ | | |
|  | -0.73 _ID.emotiondisgusted_ | | | -0.73 _ID.emotiondisgusted_ | | | -0.73 _ID.emotiondisgusted_ | | | -0.73 _ID.emotiondisgusted_ | | |
|  | -0.70 _ID.emotionafraid_ | | | -0.70 _ID.emotionafraid_ | | | -0.70 _ID.emotionafraid_ | | | -0.70 _ID.emotionafraid_ | | |
|  | -0.82 _ID.emotionjoyful_ | | | -0.82 _ID.emotionjoyful_ | | | -0.82 _ID.emotionjoyful_ | | | -0.82 _ID.emotionjoyful_ | | |
| N | 99 _ID_ | | | 99 _ID_ | | | 99 _ID_ | | | 99 _ID_ | | |
|  | 66 _video_ | | | 66 _video_ | | | 66 _video_ | | | 66 _video_ | | |
| Observations | 5843 | | | 5843 | | | 5843 | | | 5843 | | |
| Marginal R^2^ / Conditional R^2^ | 0.061 / 0.477 | | | 0.061 / 0.477 | | | 0.061 / 0.477 | | | 0.061 / 0.477 | | |

**S3.2 Predicting emotional specificity**

Creating specificity as the difference between the target-emotion rating and the next highest rating on another emotion. Specificity is greater than 0 if the addressed emotion is the highest rated emotion on average. It is smaller than 0 if at least one other emotion was rated higher on average. It is zero if the target emotion is rated highest but at least one alternative emotion is rated equally high.

# (targetemorating - maxnontarget)

D_FE1 %<>%

mutate(spec.new = case_when(

emotion == 'afraid' ~ targetemorating - select(.data = D_FE1, "sad", "joyful", "disgusted", "angry", "amused", "surprised", "shamed", "neutral") %>% do.call(pmax,.),

emotion == 'angry' ~ targetemorating - select(.data = D_FE1, "sad", "joyful", "disgusted", "afraid", "amused", "surprised", "shamed", "neutral") %>% do.call(pmax,.),

emotion == 'disgusted' ~ targetemorating - select(.data = D_FE1, "sad", "joyful", "afraid", "angry", "amused", "surprised", "shamed", "neutral") %>% do.call(pmax,.),

emotion == 'joyful' ~ targetemorating - select(.data = D_FE1, "sad", "disgusted", "afraid", "angry", "amused", "surprised", "shamed", "neutral") %>% do.call(pmax,.),

emotion == 'neutral' ~ targetemorating - select(.data = D_FE1, "sad", "joyful", "disgusted", "afraid", "angry", "amused", "surprised", "shamed") %>% do.call(pmax,.),

emotion == 'sad' ~ targetemorating - select(.data = D_FE1, "joyful", "disgusted", "afraid", "angry", "amused", "surprised", "shamed", "neutral") %>% do.call(pmax,.)

))

## Model specification and estimation

The dependent variable is the specificity of emotional responding, called spec.new.
The model is repeated four times with neutral as reference category throughout all models. However, the dummy codes for age group are recoded, making each age group the reference group in one of the models.

The intercept represents the mean specificity for *neutral* film clips for the age group that serves as reference group. The main effects for the *age contrasts* are the difference between the mean of the contrasted group and the reference group (*contrast group - reference group*) on the *neutral* rating. The main effect of the *emotion* is the difference between the mean of the respective emotion and the mean of the neutral condition, both across all age groups. The interaction terms indicate that the specificity for the respective emotion differ in the respective age group, compared to the specificity of the *neutral* condition.

## Specification of all models in ascending order of complexity - age contrast, compare base group with each other - intercept is grand mean across all age groups in neutral - emotion parameters are deviation of respective emotion grand mean from grand mean neutral - Interactions are deviation reference group vs. each other group compared ##

M0.Spec = 'spec.new ~ 1 + (1|ID) + (1|video)'

M1.Spec = 'spec.new ~ agegroup+emotion + (1|ID) + (1|video)'

M2.Spec = 'spec.new ~ agegroup+emotion+agegroup:emotion + (1|ID) + (1|video)'

M3.Spec = 'spec.new ~ agegroup+emotion+agegroup:emotion + (1+emotion|ID) + (1|video)'

M4.Spec = 'spec.new ~ agegroup+emotion+agegroup:emotion + (1+emotion|ID) + (1+agegroup|video)'

## set contrast 1 - Older. vs all + neutral vs. all ##

D_FE1$agegroup %<>% factor(levels=c("old_ad","middle_ad","young_ad","teen"))

contrasts(D_FE1$agegroup) = matrix(c(0,1,0,0,

0,0,1,0,

0,0,0,1),nrow=4,ncol=3)

dimnames(contrasts(D_FE1$agegroup))[[2]] = c("Older_vs_middle","Older_vs_Younger","Older_vs_teen")

## neutral vs. all

contrasts(D_FE1$emotion) = matrix(c(0,1,0,0,0,0,

0,0,1,0,0,0,

0,0,0,1,0,0,

0,0,0,0,1,0,

0,0,0,0,0,1),nrow=6,ncol=5)

dimnames(contrasts(D_FE1$emotion))[[2]] = c("neutral_vs_anger","neutral_vs_disgust","neutral_vs_fear","neutral_vs_happiness","neutral_vs_sadness")

Fit0.Spec.Older.neutral = lmer(M0.Spec,D_FE1,REML=TRUE,na.action="na.exclude")

Fit1.Spec.Older.neutral = lmer(M1.Spec,D_FE1,REML = TRUE,na.action = "na.exclude")

Fit2.Spec.Older.neutral = lmer(M2.Spec,D_FE1,REML = TRUE,na.action = "na.exclude")

Fit3.Spec.Older.neutral = lmer(M3.Spec,D_FE1,REML = TRUE,na.action = "na.exclude")

Fit4.Spec.Older.neutral = lmer(M4.Spec,D_FE1,REML = TRUE,na.action = "na.exclude")

## set contrast 2 - Older. vs all + anger vs. all ##

contrasts(D_FE1$emotion) = matrix(c(1,0,0,0,0,0,

0,0,1,0,0,0,

0,0,0,1,0,0,

0,0,0,0,1,0,

0,0,0,0,0,1),nrow=6,ncol=5)

dimnames(contrasts(D_FE1$emotion))[[2]] = c("anger_vs_neutral","anger_vs_disgust","anger_vs_fear","anger_vs_happiness","anger_vs_sadness")

Fit0.Spec.Older.anger = lmer(M0.Spec,D_FE1,REML=TRUE,na.action="na.exclude")

Fit1.Spec.Older.anger = lmer(M1.Spec,D_FE1,REML = TRUE,na.action = "na.exclude")

Fit2.Spec.Older.anger = lmer(M2.Spec,D_FE1,REML = TRUE,na.action = "na.exclude")

Fit3.Spec.Older.anger = lmer(M3.Spec,D_FE1,REML = TRUE,na.action = "na.exclude")

Fit4.Spec.Older.anger = lmer(M4.Spec,D_FE1,REML = TRUE,na.action = "na.exclude")

## set contrast 3 - Older. vs all + disgust vs. all ##

contrasts(D_FE1$emotion) = matrix(c(1,0,0,0,0,0,

0,1,0,0,0,0,

0,0,0,1,0,0,

0,0,0,0,1,0,

0,0,0,0,0,1),nrow=6,ncol=5)

dimnames(contrasts(D_FE1$emotion))[[2]] = c("disgust_vs_neutral","disgust_vs_anger","disgust_vs_fear","disgust_vs_happiness","disgust_vs_sadness")

Fit0.Spec.Older.disgust = lmer(M0.Spec,D_FE1,REML=TRUE,na.action="na.exclude")

Fit1.Spec.Older.disgust = lmer(M1.Spec,D_FE1,REML = TRUE,na.action = "na.exclude")

Fit2.Spec.Older.disgust = lmer(M2.Spec,D_FE1,REML = TRUE,na.action = "na.exclude")

Fit3.Spec.Older.disgust = lmer(M3.Spec,D_FE1,REML = TRUE,na.action = "na.exclude")

Fit4.Spec.Older.disgust = lmer(M4.Spec,D_FE1,REML = TRUE,na.action = "na.exclude")

## set contrast 4 - Older. vs all + fear vs. all ##

contrasts(D_FE1$emotion) = matrix(c(1,0,0,0,0,0,

0,1,0,0,0,0,

0,0,1,0,0,0,

0,0,0,0,1,0,

0,0,0,0,0,1),nrow=6,ncol=5)

dimnames(contrasts(D_FE1$emotion))[[2]] = c("fear_vs_neutral","fear_vs_anger","fear_vs_disgust","fear_vs_happiness","fear_vs_sadness")

Fit0.Spec.Older.fear = lmer(M0.Spec,D_FE1,REML=TRUE,na.action="na.exclude")

Fit1.Spec.Older.fear = lmer(M1.Spec,D_FE1,REML = TRUE,na.action = "na.exclude")

Fit2.Spec.Older.fear = lmer(M2.Spec,D_FE1,REML = TRUE,na.action = "na.exclude")

Fit3.Spec.Older.fear = lmer(M3.Spec,D_FE1,REML = TRUE,na.action = "na.exclude")

Fit4.Spec.Older.fear = lmer(M4.Spec,D_FE1,REML = TRUE,na.action = "na.exclude")

## set contrast 5 - Older. vs all + happiness vs. all ##

contrasts(D_FE1$emotion) = matrix(c(1,0,0,0,0,0,

0,1,0,0,0,0,

0,0,1,0,0,0,

0,0,0,1,0,0,

0,0,0,0,0,1),nrow=6,ncol=5)

dimnames(contrasts(D_FE1$emotion))[[2]] = c("happiness_vs_neutral","happiness_vs_anger","happiness_vs_disgust","happiness_vs_fear","happiness_vs_sadness")

Fit0.Spec.Older.happiness = lmer(M0.Spec,D_FE1,REML=TRUE,na.action="na.exclude")

Fit1.Spec.Older.happiness = lmer(M1.Spec,D_FE1,REML = TRUE,na.action = "na.exclude")

Fit2.Spec.Older.happiness = lmer(M2.Spec,D_FE1,REML = TRUE,na.action = "na.exclude")

Fit3.Spec.Older.happiness = lmer(M3.Spec,D_FE1,REML = TRUE,na.action = "na.exclude")

Fit4.Spec.Older.happiness = lmer(M4.Spec,D_FE1,REML = TRUE,na.action = "na.exclude")

## set contrast 6 - Older. vs all + anger vs. all ##

contrasts(D_FE1$emotion) = matrix(c(1,0,0,0,0,0,

0,1,0,0,0,0,

0,0,1,0,0,0,

0,0,0,1,0,0,

0,0,0,0,1,0),nrow=6,ncol=5)

dimnames(contrasts(D_FE1$emotion))[[2]] = c("sadness_vs_neutral","sadness_vs_anger","sadness_vs_disgust","sadness_vs_fear","sadness_vs_happiness")

Fit0.Spec.Older.sadness = lmer(M0.Spec,D_FE1,REML=TRUE,na.action="na.exclude")

Fit1.Spec.Older.sadness = lmer(M1.Spec,D_FE1,REML = TRUE,na.action = "na.exclude")

Fit2.Spec.Older.sadness = lmer(M2.Spec,D_FE1,REML = TRUE,na.action = "na.exclude")

Fit3.Spec.Older.sadness = lmer(M3.Spec,D_FE1,REML = TRUE,na.action = "na.exclude")

Fit4.Spec.Older.sadness = lmer(M4.Spec,D_FE1,REML = TRUE,na.action = "na.exclude")

## Model selection

Checking for singularity of models. Singularity indicates problems with parameter estimation, often arising from high collinearity or correlation among the predictors. To address this issue, we made the decision to exclude models that consistently yielded singularity across all defined contrasts. However, we adopted a slightly different approach when only one specific defined contrast led to singularity. In such cases, we exercised caution but decided to retain the model, considering the possibility that singularity may be driven by unique characteristics or relationships captured by that contrast. In the case of a retained model with a singular fit, this is additionally mentioned after the result tables. For the remaining models, likelihood-ratio-test indicate which terms are meaningful.

### check singularity of models ###

Fit4.Spec.Older.neutral %>% isSingular()

## [1] TRUE

Fit4.Spec.Older.anger %>% isSingular()

## [1] TRUE

Fit4.Spec.Older.disgust %>% isSingular()

## [1] TRUE

Fit4.Spec.Older.fear %>% isSingular()

## [1] TRUE

Fit4.Spec.Older.happiness %>% isSingular()

## [1] TRUE

Fit4.Spec.Older.sadness %>% isSingular()

## [1] TRUE

# Model 4 is singular in all cases

Fit3.Spec.Older.neutral %>% isSingular()

## [1] FALSE

Fit3.Spec.Older.anger %>% isSingular()

## [1] FALSE

Fit3.Spec.Older.disgust %>% isSingular()# is singular, but only with disgust as reference (and our default is neutral).

## [1] TRUE

Fit3.Spec.Older.fear %>% isSingular()

## [1] FALSE

Fit3.Spec.Older.happiness %>% isSingular()

## [1] FALSE

Fit3.Spec.Older.sadness %>% isSingular()

## [1] FALSE

### check model improvement ###

lrtest(Fit3.Spec.Older.neutral,

Fit2.Spec.Older.neutral,

Fit1.Spec.Older.neutral,

Fit0.Spec.Older.neutral)

## Likelihood ratio test

##

## Model 1: spec.new ~ agegroup + emotion + agegroup:emotion + (1 + emotion |

## ID) + (1 | video)

## Model 2: spec.new ~ agegroup + emotion + agegroup:emotion + (1 | ID) +

## (1 | video)

## Model 3: spec.new ~ agegroup + emotion + (1 | ID) + (1 | video)

## Model 4: spec.new ~ 1 + (1 | ID) + (1 | video)

## #Df LogLik Df Chisq Pr(>Chisq)

## 1 46 -13428

## 2 26 -13806 -20 757.144 < 2.2e-16 ***

## 3 12 -13874 -14 134.828 < 2.2e-16 ***

## 4 4 -13904 -8 60.702 3.394e-10 ***

## ---

## Signif. codes: 0 '***' 0.001 '**' 0.01 '*' 0.05 '.' 0.1 ' ' 1

## significance of random effects

ranova(Fit3.Spec.Older.neutral)

## ANOVA-like table for random-effects: Single term deletions

##

## Model:

## spec.new ~ agegroup + emotion + (1 + emotion | ID) + (1 | video) + agegroup:emotion

## npar logLik AIC LRT Df Pr(>Chisq)

## <none> 46 -13428 26948

## emotion in (1 + emotion | ID) 26 -13806 27665 757.14 20 < 2.2e-16 ***

## (1 | video) 45 -13645 27380 434.75 1 < 2.2e-16 ***

## ---

## Signif. codes: 0 '***' 0.001 '**' 0.01 '*' 0.05 '.' 0.1 ' ' 1

It follows that the model spec.new ~ agegroup+emotion+agegroup:emotion + (1+emotion|ID) + (1|video) fits best. Even if the correlation between some of the random intercepts and slopes are nearly -1/1 (presented in the following results table) and a singularity could be assumed.

## Results table for emotional specificity

## Creates an HTML table for the model. Outputs additional information, such as p-values and confidence intervals. ##

tab_model(Fit3.Spec.Older.neutral,

Fit3.Spec.Older.anger,

Fit3.Spec.Older.disgust,

Fit3.Spec.Older.fear,

Fit3.Spec.Older.happiness,

Fit3.Spec.Older.sadness,

dv.labels = c('Reference: Neutral',

'Reference: Anger',

'Reference: Disgust',

'Reference: Fear',

'Reference: Happiness',

'Reference: Sadness'),

show.re.var = T,

show.icc = F)

|  | **Reference: Neutral** | | | **Reference: Sad** | | | **Reference: Angry** | | | **Reference: Disgusted** | | | **Reference: Afraid** | | | **Reference: Joyful** | | |
| --- | --- | --- | --- | --- | --- | --- | --- | --- | --- | --- | --- | --- | --- | --- | --- | --- | --- | --- |
| *Predictors* | *Esti-mates* | *CI* | *p* | *Esti-mates* | *CI* | *p* | *Esti-mates* | *CI* | *p* | *Esti-mates* | *CI* | *p* | *Esti-mates* | *CI* | *p* | *Esti-mates* | *CI* | *p* |
| (Intercept) | 1.02 | 0.05 – 1.98 | **0.038** | 1.00 | 0.34 – 1.65 | **0.003** | -0.28 | -0.97 – 0.40 | 0.417 | -0.09 | -0.89 – 0.71 | 0.820 | -0.40 | -1.24 – 0.44 | 0.352 | 0.19 | -0.47 – 0.86 | 0.570 |
| agegroup older vs middle | 1.26 | 0.03 – 2.50 | **0.045** | 0.43 | -0.33 – 1.19 | 0.266 | 0.31 | -0.42 – 1.03 | 0.408 | 0.59 | -0.37 – 1.56 | 0.230 | 0.75 | -0.31 – 1.82 | 0.166 | -0.34 | -0.98 – 0.30 | 0.292 |
| agegroup older vs Younger | 2.06 | 0.88 – 3.24 | **0.001** | 0.20 | -0.52 – 0.93 | 0.583 | 0.20 | -0.50 – 0.90 | 0.572 | 1.39 | 0.47 – 2.32 | **0.003** | 0.22 | -0.80 – 1.23 | 0.677 | -0.77 | -1.38 – -0.15 | **0.014** |
| agegroup older vs teen | 2.08 | 0.93 – 3.23 | **<0.001** | 0.63 | -0.09 – 1.36 | 0.087 | -0.35 | -1.07 – 0.37 | 0.341 | 1.77 | 0.85 – 2.69 | **<0.001** | -0.36 | -0.95 – 0.24 | 0.240 | -0.36 | -0.95 – 0.24 | 0.240 |
| emotion neutral vs sad | -0.02 | -1.16 – 1.12 | 0.972 |  |  |  |  |  |  |  |  |  |  |  |  |  |  |  |
| emotion neutral vs angry | -1.30 | -2.47 – -0.13 | **0.030** |  |  |  |  |  |  |  |  |  |  |  |  |  |  |  |
| emotion neutral vs disgusted | -1.11 | -2.37 – 0.15 | 0.085 |  |  |  |  |  |  |  |  |  |  |  |  |  |  |  |
| emotion neutral vs afraid | -1.42 | -2.69 – -0.15 | **0.029** |  |  |  |  |  |  |  |  |  |  |  |  |  |  |  |
| emotion neutral vs joyful | -0.82 | -2.06 – 0.41 | 0.192 |  |  |  |  |  |  |  |  |  |  |  |  |  |  |  |
| agegroup older vs middle × emotion neutral vs sad | -0.83 | -2.23 – 0.57 | 0.244 |  |  |  |  |  |  |  |  |  |  |  |  |  |  |  |
| agegroup older vs Younger × emotion neutral vs sad | -1.85 | -3.19 – -0.51 | **0.007** |  |  |  |  |  |  |  |  |  |  |  |  |  |  |  |
| agegroup older vs teen × emotion neutral vs sad | -1.45 | -2.76 – -0.14 | **0.031** |  |  |  |  |  |  |  |  |  |  |  |  |  |  |  |
| agegroup older vs middle × emotion neutral vs angry | -0.96 | -2.38 – 0.46 | 0.184 |  |  |  |  |  |  |  |  |  |  |  |  |  |  |  |
| agegroup older vs Younger × emotion neutral vs angry | -1.85 | -3.21 – -0.50 | **0.007** |  |  |  |  |  |  |  |  |  |  |  |  |  |  |  |
| agegroup older vs teen × emotion neutral vs angry | -2.43 | -3.77 – -1.09 | **<0.001** |  |  |  |  |  |  |  |  |  |  |  |  |  |  |  |
| agegroup older vs middle × emotion neutral vs disgusted | -0.67 | -2.26 – 0.92 | 0.408 |  |  |  |  |  |  |  |  |  |  |  |  |  |  |  |
| agegroup older vs Younger × emotion neutral vs disgusted | -0.67 | -2.18 – 0.85 | 0.391 |  |  |  |  |  |  |  |  |  |  |  |  |  |  |  |
| agegroup older vs teen × emotion neutral vs disgusted | -0.31 | -1.80 – 1.18 | 0.682 |  |  |  |  |  |  |  |  |  |  |  |  |  |  |  |
| agegroup older vs middle × emotion neutral vs afraid | -0.51 | -2.12 – 1.10 | 0.535 |  |  |  |  |  |  |  |  |  |  |  |  |  |  |  |
| agegroup older vs Younger × emotion neutral vs afraid | -1.84 | -3.38 – -0.30 | **0.019** |  |  |  |  |  |  |  |  |  |  |  |  |  |  |  |
| agegroup older vs middle × emotion neutral vs joyful | -1.61 | -3.12 – -0.10 | **0.037** |  |  |  |  |  |  |  |  |  |  |  |  |  |  |  |
| agegroup older vs Younger × emotion neutral vs joyful | -2.82 | -4.27 – -1.38 | **<0.001** |  |  |  |  |  |  |  |  |  |  |  |  |  |  |  |
| agegroup older vs teen × emotion neutral vs joyful | -2.44 | -3.84 – -1.03 | **0.001** |  |  |  |  |  |  |  |  |  |  |  |  |  |  |  |
| emotion sad vs neutral |  |  |  | 0.02 | -1.12 – 1.16 | 0.972 |  |  |  |  |  |  |  |  |  |  |  |  |
| emotion sad vs angry |  |  |  | -1.28 | -2.12 – -0.44 | **0.003** |  |  |  |  |  |  |  |  |  |  |  |  |
| emotion sad vs disgusted |  |  |  | -1.09 | -1.99 – -0.19 | **0.017** |  |  |  |  |  |  |  |  |  |  |  |  |
| emotion sad vs afraid |  |  |  | -1.40 | -2.26 – -0.53 | **0.001** |  |  |  |  |  |  |  |  |  |  |  |  |
| emotion sad vs joyful |  |  |  | -0.80 | -1.62 – 0.02 | 0.055 |  |  |  |  |  |  |  |  |  |  |  |  |
| agegroup older vs middle × emotion sad vs neutral |  |  |  | 0.83 | -0.57 – 2.23 | 0.244 |  |  |  |  |  |  |  |  |  |  |  |  |
| agegroup older vs Younger × emotion sad vs neutral |  |  |  | 1.85 | 0.51 – 3.19 | **0.007** |  |  |  |  |  |  |  |  |  |  |  |  |
| agegroup older vs teen × emotion sad vs neutral |  |  |  | 1.45 | 0.14 – 2.76 | **0.031** |  |  |  |  |  |  |  |  |  |  |  |  |
| agegroup older vs middle × emotion sad vs angry |  |  |  | -0.13 | -0.95 – 0.69 | 0.758 |  |  |  |  |  |  |  |  |  |  |  |  |
| agegroup older vs Younger × emotion sad vs angry |  |  |  | -0.00 | -0.78 – 0.78 | 0.998 |  |  |  |  |  |  |  |  |  |  |  |  |
| agegroup older vs teen × emotion sad vs angry |  |  |  | -0.98 | -1.80 – -0.17 | **0.018** |  |  |  |  |  |  |  |  |  |  |  |  |
| agegroup older vs middle × emotion sad vs disgusted |  |  |  | 0.16 | -0.81 – 1.13 | 0.745 |  |  |  |  |  |  |  |  |  |  |  |  |
| agegroup older vs Younger × emotion sad vs disgusted |  |  |  | 1.19 | 0.26 – 2.12 | **0.012** |  |  |  |  |  |  |  |  |  |  |  |  |
| agegroup older vs teen × emotion sad vs disgusted |  |  |  | 1.14 | 0.20 – 2.07 | **0.017** |  |  |  |  |  |  |  |  |  |  |  |  |
| agegroup older vs middle × emotion sad vs afraid |  |  |  | 0.32 | -0.60 – 1.24 | 0.494 |  |  |  |  |  |  |  |  |  |  |  |  |
| agegroup older vs Younger × emotion sad vs afraid |  |  |  | 0.01 | -0.87 – 0.89 | 0.978 |  |  |  |  |  |  |  |  |  |  |  |  |
| agegroup older vs middle × emotion sad vs joyful |  |  |  | -0.78 | -1.52 – -0.03 | **0.041** |  |  |  |  |  |  |  |  |  |  |  |  |
| agegroup older vs Younger × emotion sad vs joyful |  |  |  | -0.97 | -1.68 – -0.26 | **0.008** |  |  |  |  |  |  |  |  |  |  |  |  |
| agegroup older vs teen × emotion sad vs joyful |  |  |  | -0.99 | -1.70 – -0.28 | **0.006** |  |  |  |  |  |  |  |  |  |  |  |  |
| emotion angry vs neutral |  |  |  |  |  |  | 1.30 | 0.17 – 2.43 | **0.024** |  |  |  |  |  |  |  |  |  |
| emotion angry vs sad |  |  |  |  |  |  | 1.28 | 0.55 – 2.01 | **0.001** |  |  |  |  |  |  |  |  |  |
| emotion angry vs disgusted |  |  |  |  |  |  | 0.19 | -0.76 – 1.14 | 0.693 |  |  |  |  |  |  |  |  |  |
| emotion angry vs afraid |  |  |  |  |  |  | -0.12 | -1.04 – 0.80 | 0.805 |  |  |  |  |  |  |  |  |  |
| emotion angry vs joyful |  |  |  |  |  |  | 0.48 | -0.40 – 1.36 | 0.288 |  |  |  |  |  |  |  |  |  |
| agegroup older vs middle × emotion angry vs neutral |  |  |  |  |  |  | 0.96 | -0.38 – 2.29 | 0.162 |  |  |  |  |  |  |  |  |  |
| agegroup older vs Younger × emotion angry vs neutral |  |  |  |  |  |  | 1.86 | 0.58 – 3.14 | **0.004** |  |  |  |  |  |  |  |  |  |
| agegroup older vs teen × emotion angry vs neutral |  |  |  |  |  |  | 2.43 | 1.16 – 3.70 | **<0.001** |  |  |  |  |  |  |  |  |  |
| agegroup older vs middle × emotion angry vs sad |  |  |  |  |  |  | 0.12 | -0.43 – 0.68 | 0.666 |  |  |  |  |  |  |  |  |  |
| agegroup older vs Younger × emotion angry vs sad |  |  |  |  |  |  | 0.00 | -0.53 – 0.54 | 0.987 |  |  |  |  |  |  |  |  |  |
| agegroup older vs teen × emotion angry vs sad |  |  |  |  |  |  | 0.98 | 0.39 – 1.58 | **0.001** |  |  |  |  |  |  |  |  |  |
| agegroup older vs middle × emotion angry vs disgusted |  |  |  |  |  |  | 0.28 | -0.72 – 1.29 | 0.580 |  |  |  |  |  |  |  |  |  |
| agegroup older vs Younger × emotion angry vs disgusted |  |  |  |  |  |  | 1.19 | 0.23 – 2.16 | **0.016** |  |  |  |  |  |  |  |  |  |
| agegroup older vs teen × emotion angry vs disgusted |  |  |  |  |  |  | 2.12 | 1.13 – 3.11 | **<0.001** |  |  |  |  |  |  |  |  |  |
| agegroup older vs middle × emotion angry vs afraid |  |  |  |  |  |  | 0.44 | -0.52 – 1.41 | 0.367 |  |  |  |  |  |  |  |  |  |
| agegroup older vs Younger × emotion angry vs afraid |  |  |  |  |  |  | 0.02 | -0.91 – 0.94 | 0.972 |  |  |  |  |  |  |  |  |  |
| agegroup older vs middle × emotion angry vs joyful |  |  |  |  |  |  | -0.65 | -1.45 – 0.14 | 0.107 |  |  |  |  |  |  |  |  |  |
| agegroup older vs Younger × emotion angry vs joyful |  |  |  |  |  |  | -0.96 | -1.73 – -0.20 | **0.013** |  |  |  |  |  |  |  |  |  |
| agegroup older vs teen × emotion angry vs joyful |  |  |  |  |  |  | -0.01 | -0.79 – 0.77 | 0.989 |  |  |  |  |  |  |  |  |  |
| emotion disgusted vs neutral |  |  |  |  |  |  |  |  |  | 1.11 | -0.15 – 2.37 | 0.085 |  |  |  |  |  |  |
| emotion disgusted vs sad |  |  |  |  |  |  |  |  |  | 1.09 | 0.19 – 1.99 | **0.017** |  |  |  |  |  |  |
| emotion disgusted vs angry |  |  |  |  |  |  |  |  |  | -0.19 | -1.22 – 0.83 | 0.714 |  |  |  |  |  |  |
| emotion disgusted vs afraid |  |  |  |  |  |  |  |  |  | -0.31 | -1.24 – 0.63 | 0.519 |  |  |  |  |  |  |
| emotion disgusted vs joyful |  |  |  |  |  |  |  |  |  | 0.29 | -0.67 – 1.24 | 0.558 |  |  |  |  |  |  |
| agegroup older vs middle × emotion disgusted vs neutral |  |  |  |  |  |  |  |  |  | 0.67 | -0.92 – 2.26 | 0.408 |  |  |  |  |  |  |
| agegroup older vs Younger × emotion disgusted vs neutral |  |  |  |  |  |  |  |  |  | 0.67 | -0.85 – 2.18 | 0.391 |  |  |  |  |  |  |
| agegroup older vs teen × emotion disgusted vs neutral |  |  |  |  |  |  |  |  |  | 0.31 | -1.18 – 1.80 | 0.682 |  |  |  |  |  |  |
| agegroup older vs middle × emotion disgusted vs sad |  |  |  |  |  |  |  |  |  | -0.16 | -1.13 – 0.81 | 0.745 |  |  |  |  |  |  |
| agegroup older vs Younger × emotion disgusted vs sad |  |  |  |  |  |  |  |  |  | -1.19 | -2.12 – -0.26 | **0.012** |  |  |  |  |  |  |
| agegroup older vs teen × emotion disgusted vs sad |  |  |  |  |  |  |  |  |  | -1.14 | -2.07 – -0.20 | **0.017** |  |  |  |  |  |  |
| agegroup older vs middle × emotion disgusted vs angry |  |  |  |  |  |  |  |  |  | -0.29 | -1.44 – 0.87 | 0.623 |  |  |  |  |  |  |
| agegroup older vs Younger × emotion disgusted vs angry |  |  |  |  |  |  |  |  |  | -1.19 | -2.29 – -0.08 | **0.035** |  |  |  |  |  |  |
| agegroup older vs teen × emotion disgusted vs angry |  |  |  |  |  |  |  |  |  | -2.12 | -3.24 – -1.01 | **<0.001** |  |  |  |  |  |  |
| agegroup older vs middle × emotion disgusted vs afraid |  |  |  |  |  |  |  |  |  | 0.16 | -0.86 – 1.18 | 0.759 |  |  |  |  |  |  |
| agegroup older vs Younger × emotion disgusted vs afraid |  |  |  |  |  |  |  |  |  | -1.18 | -2.15 – -0.20 | **0.018** |  |  |  |  |  |  |
| agegroup older vs middle × emotion disgusted vs joyful |  |  |  |  |  |  |  |  |  | -0.94 | -1.93 – 0.05 | 0.064 |  |  |  |  |  |  |
| agegroup older vs Younger × emotion disgusted vs joyful |  |  |  |  |  |  |  |  |  | -2.16 | -3.11 – -1.21 | **<0.001** |  |  |  |  |  |  |
| agegroup older vs teen × emotion disgusted vs joyful |  |  |  |  |  |  |  |  |  | -2.13 | -3.06 – -1.19 | **<0.001** |  |  |  |  |  |  |
| emotion afraid vs neutral |  |  |  |  |  |  |  |  |  |  |  |  | 1.42 | 0.15 – 2.68 | **0.029** |  |  |  |
| emotion afraid vs sad |  |  |  |  |  |  |  |  |  |  |  |  | 1.40 | 0.53 – 2.26 | **0.001** |  |  |  |
| emotion afraid vs angry |  |  |  |  |  |  |  |  |  |  |  |  | 0.12 | -0.88 – 1.11 | 0.819 |  |  |  |
| emotion afraid vs disgusted |  |  |  |  |  |  |  |  |  |  |  |  | 0.31 | -0.63 – 1.24 | 0.519 |  |  |  |
| emotion afraid vs joyful |  |  |  |  |  |  |  |  |  |  |  |  | 0.59 | -0.39 – 1.57 | 0.236 |  |  |  |
| agegroup older vs middle × emotion afraid vs neutral |  |  |  |  |  |  |  |  |  |  |  |  | 0.51 | -1.10 – 2.12 | 0.535 |  |  |  |
| agegroup older vs Younger × emotion afraid vs neutral |  |  |  |  |  |  |  |  |  |  |  |  | 1.84 | 0.30 – 3.38 | **0.019** |  |  |  |
| agegroup older vs teen × emotion afraid vs neutral |  |  |  |  |  |  |  |  |  |  |  |  | 2.44 | 1.03 – 3.84 | **0.001** |  |  |  |
| agegroup older vs middle × emotion afraid vs sad |  |  |  |  |  |  |  |  |  |  |  |  | -0.32 | -1.24 – 0.60 | 0.494 |  |  |  |
| agegroup older vs Younger × emotion afraid vs sad |  |  |  |  |  |  |  |  |  |  |  |  | -0.01 | -0.89 – 0.87 | 0.978 |  |  |  |
| agegroup older vs teen × emotion afraid vs sad |  |  |  |  |  |  |  |  |  |  |  |  | 0.99 | 0.28 – 1.70 | **0.006** |  |  |  |
| agegroup older vs middle × emotion afraid vs angry |  |  |  |  |  |  |  |  |  |  |  |  | -0.45 | -1.57 – 0.67 | 0.431 |  |  |  |
| agegroup older vs Younger × emotion afraid vs angry |  |  |  |  |  |  |  |  |  |  |  |  | -0.01 | -1.08 – 1.06 | 0.981 |  |  |  |
| agegroup older vs teen × emotion afraid vs angry |  |  |  |  |  |  |  |  |  |  |  |  | 0.00 | -0.93 – 0.94 | 0.992 |  |  |  |
| agegroup older vs middle × emotion afraid vs disgusted |  |  |  |  |  |  |  |  |  |  |  |  | -0.16 | -1.18 – 0.86 | 0.759 |  |  |  |
| agegroup older vs Younger × emotion afraid vs disgusted |  |  |  |  |  |  |  |  |  |  |  |  | 1.18 | 0.20 – 2.15 | **0.018** |  |  |  |
| agegroup older vs teen × emotion afraid vs disgusted |  |  |  |  |  |  |  |  |  |  |  |  | 2.13 | 1.19 – 3.06 | **<0.001** |  |  |  |
| agegroup older vs middle × emotion afraid vs joyful |  |  |  |  |  |  |  |  |  |  |  |  | -1.10 | -2.16 – -0.04 | **0.043** |  |  |  |
| agegroup older vs Younger × emotion afraid vs joyful |  |  |  |  |  |  |  |  |  |  |  |  | -0.98 | -2.00 – 0.03 | 0.058 |  |  |  |
| emotion joyful vs neutral |  |  |  |  |  |  |  |  |  |  |  |  |  |  |  | 0.82 | -0.41 – 2.06 | 0.192 |
| emotion joyful vs sad |  |  |  |  |  |  |  |  |  |  |  |  |  |  |  | 0.80 | -0.02 – 1.62 | 0.055 |
| emotion joyful vs angry |  |  |  |  |  |  |  |  |  |  |  |  |  |  |  | -0.48 | -1.43 – 0.48 | 0.328 |
| emotion joyful vs disgusted |  |  |  |  |  |  |  |  |  |  |  |  |  |  |  | -0.29 | -1.24 – 0.67 | 0.558 |
| emotion joyful vs afraid |  |  |  |  |  |  |  |  |  |  |  |  |  |  |  | -0.59 | -1.57 – 0.39 | 0.236 |
| agegroup older vs middle × emotion joyful vs neutral |  |  |  |  |  |  |  |  |  |  |  |  |  |  |  | 1.61 | 0.10 – 3.12 | **0.037** |
| agegroup older vs Younger × emotion joyful vs neutral |  |  |  |  |  |  |  |  |  |  |  |  |  |  |  | 2.82 | 1.38 – 4.27 | **<0.001** |
| agegroup older vs teen × emotion joyful vs neutral |  |  |  |  |  |  |  |  |  |  |  |  |  |  |  | 2.44 | 1.03 – 3.84 | **0.001** |
| agegroup older vs middle × emotion joyful vs sad |  |  |  |  |  |  |  |  |  |  |  |  |  |  |  | 0.78 | 0.03 – 1.52 | **0.041** |
| agegroup older vs Younger × emotion joyful vs sad |  |  |  |  |  |  |  |  |  |  |  |  |  |  |  | 0.97 | 0.26 – 1.68 | **0.008** |
| agegroup older vs teen × emotion joyful vs sad |  |  |  |  |  |  |  |  |  |  |  |  |  |  |  | 0.99 | 0.28 – 1.70 | **0.006** |
| agegroup older vs middle × emotion joyful vs angry |  |  |  |  |  |  |  |  |  |  |  |  |  |  |  | 0.65 | -0.32 – 1.62 | 0.191 |
| agegroup older vs Younger × emotion joyful vs angry |  |  |  |  |  |  |  |  |  |  |  |  |  |  |  | 0.97 | 0.04 – 1.90 | **0.041** |
| agegroup older vs teen × emotion joyful vs angry |  |  |  |  |  |  |  |  |  |  |  |  |  |  |  | 0.00 | -0.93 – 0.94 | 0.992 |
| agegroup older vs middle × emotion joyful vs disgusted |  |  |  |  |  |  |  |  |  |  |  |  |  |  |  | 0.94 | -0.05 – 1.93 | 0.064 |
| agegroup older vs Younger × emotion joyful vs disgusted |  |  |  |  |  |  |  |  |  |  |  |  |  |  |  | 2.16 | 1.21 – 3.11 | **<0.001** |
| agegroup older vs teen × emotion joyful vs disgusted |  |  |  |  |  |  |  |  |  |  |  |  |  |  |  | 2.13 | 1.19 – 3.06 | **<0.001** |
| agegroup older vs middle × emotion joyful vs afraid |  |  |  |  |  |  |  |  |  |  |  |  |  |  |  | 1.10 | 0.04 – 2.16 | **0.043** |
| agegroup older vs Younger × emotion joyful vs afraid |  |  |  |  |  |  |  |  |  |  |  |  |  |  |  | 0.98 | -0.03 – 2.00 | 0.058 |
| **Random Effects** | | | | | | | | | | | | | | | | | | |
| σ^2^ | 5.05 | | | 5.05 | | | 5.15 | | | 5.05 | | | 5.05 | | | 5.05 | | |
| τ_00_ | 4.01 _ID_ | | | 1.35 _ID_ | | | 1.05 _ID_ | | | 2.31 _ID_ | | | 2.94 _ID_ | | | 0.65 _ID_ | | |
|  | 0.60 _video_ | | | 0.60 _video_ | | | 0.60 _video_ | | | 0.60 _video_ | | | 0.60 _video_ | | | 0.60 _video_ | | |
| τ_11_ | 4.95 _ID.emotion neutral_vs_sad_ | | | 4.95 _ID.emotionsad_vs_neutral_ | | | 4.28 _ID.emotionangry_vs_neutral_ | | | 6.52 _ID.emotiondisgusted_vs_neutral_ | | | 6.80 _ID.emotionafraid_vs_neutral_ | | | 5.70 _ID.emotionjoyful_vs_neutral_ | | |
|  | 4.95 _ID.emotion neutral_vs_angry_ | | | 1.11 _ID.emotionsad_vs_angry_ | | | 0.02 _ID.emotionangry_vs_sad_ | | | 1.98 _ID.emotiondisgusted_vs_sad_ | | | 1.72 _ID.emotionafraid_vs_sad_ | | | 0.71 _ID.emotionjoyful_vs_sad_ | | |
|  | 6.52 _ID.emotion neutral_vs_disgusted_ | | | 1.98 _ID.emotionsad_vs_disgusted_ | | | 2.03 _ID.emotionangry_vs_disgusted_ | | | 2.98 _ID.emotiondisgusted_vs_angry_ | | | 2.77 _ID.emotionafraid_vs_angry_ | | | 1.72 _ID.emotionjoyful_vs_angry_ | | |
|  | 6.80 _ID.emotion neutral_vs_afraid_ | | | 1.72 _ID.emotionsad_vs_afraid_ | | | 1.82 _ID.emotionangry_vs_afraid_ | | | 2.19 _ID.emotiondisgusted_vs_afraid_ | | | 2.19 _ID.emotionafraid_vs_disgusted_ | | | 1.88 _ID.emotionjoyful_vs_disgusted_ | | |
|  | 5.70 _ID.emotion neutral_vs_joyful_ | | | 0.71 _ID.emotionsad_vs_joyful_ | | | 0.78 _ID.emotionangry_vs_joyful_ | | | 1.88 _ID.emotiondisgusted_vs_joyful_ | | | 2.35 _ID.emotionafraid_vs_joyful_ | | | 2.35 _ID.emotionjoyful_vs_afraid_ | | |
| ρ_01_ | -0.85 _ID.emotion neutral_vs_sad_ | | | -0.44 _ID.emotionsad_vs_neutral_ | | | -0.31 _ID.emotionangry_vs_neutral_ | | | -0.62 _ID.emotiondisgusted_vs_neutral_ | | | -0.64 _ID.emotionafraid_vs_neutral_ | | | -0.61 _ID.emotionjoyful_vs_neutral_ | | |
|  | -0.82 _ID.emotion neutral_vs_angry_ | | | -0.33 _ID.emotionsad_vs_angry_ | | | 0.63 _ID.emotionangry_vs_sad_ | | | -0.69 _ID.emotiondisgusted_vs_sad_ | | | -0.73 _ID.emotionafraid_vs_sad_ | | | -0.01 _ID.emotionjoyful_vs_sad_ | | |
|  | -0.80 _ID.emotion neutral_vs_disgusted_ | | | -0.31 _ID.emotionsad_vs_disgusted_ | | | -0.27 _ID.emotionangry_vs_disgusted_ | | | -0.69 _ID.emotiondisgusted_vs_angry_ | | | -0.71 _ID.emotionafraid_vs_angry_ | | | -0.34 _ID.emotionjoyful_vs_angry_ | | |
|  | -0.75 _ID.emotion neutral_vs_afraid_ | | | -0.05 _ID.emotionsad_vs_afraid_ | | | 0.03 _ID.emotionangry_vs_afraid_ | | | -0.35 _ID.emotiondisgusted_vs_afraid_ | | | -0.56 _ID.emotionafraid_vs_disgusted_ | | | -0.10 _ID.emotionjoyful_vs_disgusted_ | | |
|  | -0.95 _ID.emotion neutral_vs_joyful_ | | | -0.72 _ID.emotionsad_vs_joyful_ | | | -0.66 _ID.emotionangry_vs_joyful_ | | | -0.85 _ID.emotiondisgusted_vs_joyful_ | | | -0.88 _ID.emotionafraid_vs_joyful_ | | | -0.02 _ID.emotionjoyful_vs_afraid_ | | |
| N | 99 _ID_ | | | 99 _ID_ | | | 99 _ID_ | | | 99 _ID_ | | | 99 _ID_ | | | 99 _ID_ | | |
|  | 66 _video_ | | | 66 _video_ | | | 66 _video_ | | | 66 _video_ | | | 66 _video_ | | | 66 _video_ | | |
| Observations | 5821 | | | 5821 | | | 5821 | | | 5821 | | | 5821 | | | 5821 | | |
| Marginal R^2^ / Conditional R^2^ | 0.128 / 0.434 | | | 0.128 / 0.434 | | | 0.182 / NA | | | 0.128 / 0.434 | | | 0.128 / 0.434 | | | 0.128 / 0.434 | | |

Note. In the model with "angry" as the reference category, the random slopes for "angry_vs_neutral" and "angry_vs_sad" were highly correlated. This high correlation of the random slopes made it challenging for the model to distinguish the unique effects of these slopes that tended to influence the outcome in a similar manner. This resulted in missing conditional R² and diverging random effect estimates. These particular random effect estimates as obtained from this model should be interpreted with caution. Note that this does not affect the hypotheses test, which relied on fixed-effect estimates. These estimates are not affected because they represent the average relationship between predictors and outcome.

**S3.3 Follow-up analyses: Effects across individual film clips**

## Model specification and estimation

Mean Target-emotion rating is predicted using six separate models for each of the 6 emotions as well as neutral films. Effect coding is used for both film clips and age groups. This means that the intercept is the grand mean of targetemorating, across film clips and age groups. Each estimate is the difference for the respective predictor from the grand mean.

Each model includes a random intercept for person (ID). This captures some variance due to differences between persons.

Log-likelihood ratio test are used to choose which terms improve the fit. Inclusion of the random intercept was additionally confirmed with an ANOVA for Linear Mixed Models.
Contrasts are limited to *k*−1 per predictor so that one estimate needs to be deduced from the other estimates. For convenience, each model is complemented by an additional model with recoded constrast codes, thus providing the estimates for the missing predictor as well.

### Model Specification - video ###

M0.Video.r <- 'targetemorating ~ 1 + (1|ID)'

M1.Video.r <- 'targetemorating ~ 1 + video + (1|ID)'

M3.Video.r <- 'targetemorating ~ 1 + video + agegroup + agegroup:video + (1|ID)'

### neutral videos ###

## subset the emotion and change contrast to effect coding for emotion as well as age group ##

## it follows that the coefficients mean the change with respect to the grand mean

D_FE1.neutral <- D_FE1 %>% filter(emotion=="neutral")

D_FE1.neutral$video %<>% as.factor() %>% droplevels()

contrasts(D_FE1.neutral$video) <- contr.sum(10)

dimnames(contrasts(D_FE1.neutral$video))[[2]] <- c(55:63)

D_FE1.neutral$agegroup %<>% factor(levels=c("teen","young_ad","middle_ad","old_ad"))

contrasts(D_FE1.neutral$agegroup) <- contr.sum(4)

dimnames(contrasts(D_FE1.neutral$agegroup))[[2]] <- c("teen","young_ad","middle_ad")

# with random Intercept for ID

Fit0.Video.neutral.r <- lmer(M0.Video.r,D_FE1.neutral,na.action = "na.exclude")

Fit1.Video.neutral.r <- lmer(M1.Video.r,D_FE1.neutral,na.action = "na.exclude")

Fit3.Video.neutral.r <- lmer(M3.Video.r,D_FE1.neutral,na.action = "na.exclude")

# with changed video order

D_FE1.neutral$video %<>% relevel(ref="64") %>% droplevels()

contrasts(D_FE1.neutral$video) <-contr.sum(10)

dimnames(contrasts(D_FE1.neutral$video))[[2]] <-c(64,55:62)

Fit1.Video.neutral.r.64 <- lmer(M1.Video.r,D_FE1.neutral,REML = TRUE,na.action = "na.exclude")

### angry videos ###

D_FE1.angry <- D_FE1 %>% filter(emotion=="angry")

D_FE1.angry$video %<>% as.factor() %>% droplevels()

contrasts(D_FE1.angry$video) <- contr.sum(10)

dimnames(contrasts(D_FE1.angry$video))[[2]] <-c(13:21)

D_FE1.angry$agegroup %<>% factor(levels=c("teen","young_ad","middle_ad","old_ad"))

contrasts(D_FE1.angry$agegroup) <- contr.sum(4)

dimnames(contrasts(D_FE1.angry$agegroup))[[2]] <- c("teen","young_ad","middle_ad")

# with random Intercept for ID

Fit0.Video.angry.r <- lmer(M0.Video.r,D_FE1.angry,na.action = "na.exclude")

Fit1.Video.angry.r <- lmer(M1.Video.r,D_FE1.angry,na.action = "na.exclude")

Fit3.Video.angry.r <- lmer(M3.Video.r,D_FE1.angry,na.action = "na.exclude")

# with changed video order

D_FE1.angry$video %<>% relevel(ref="22") %>% droplevels()

contrasts(D_FE1.angry$video) <- contr.sum(10)

dimnames(contrasts(D_FE1.angry$video))[[2]] <- c(22,13:20)

# with random Intercept for ID

Fit3.Video.angry.r.22 <- lmer(M3.Video.r,D_FE1.angry,na.action = "na.exclude")

### disgusted videos ###

D_FE1.disgusted <- D_FE1 %>% filter(emotion=="disgusted")

D_FE1.disgusted$video %<>% as.factor() %>% droplevels()

contrasts(D_FE1.disgusted$video) <- contr.sum(11)

dimnames(contrasts(D_FE1.disgusted$video))[[2]] <- c(23:32)

D_FE1.disgusted$agegroup %<>% factor(levels=c("teen","young_ad","middle_ad","old_ad"))

contrasts(D_FE1.disgusted$agegroup) <- contr.sum(4)

dimnames(contrasts(D_FE1.disgusted$agegroup))[[2]] <- c("teen","young_ad","middle_ad")

# with random Intercept for ID

Fit0.Video.disgusted.r <- lmer(M0.Video.r,D_FE1.disgusted,na.action = "na.exclude")

Fit1.Video.disgusted.r <- lmer(M1.Video.r,D_FE1.disgusted,na.action = "na.exclude")

Fit3.Video.disgusted.r <- lmer(M3.Video.r,D_FE1.disgusted,na.action = "na.exclude")

# with changed video order

D_FE1.disgusted$video %<>% relevel(ref="32") %>% droplevels()

contrasts(D_FE1.disgusted$video) <- contr.sum(11)

dimnames(contrasts(D_FE1.disgusted$video))[[2]] <- c(33,23:31)

# with random Intercept for ID

Fit3.Video.disgusted.r.32 <- lmer(M3.Video.r,D_FE1.disgusted,na.action = "na.exclude")

### afraid videos ###

D_FE1.afraid <- D_FE1 %>% filter(emotion=="afraid")

D_FE1.afraid$video %<>% as.factor() %>% droplevels()

contrasts(D_FE1.afraid$video) <- contr.sum(12)

dimnames(contrasts(D_FE1.afraid$video))[[2]] <- c(1:11)

D_FE1.afraid$agegroup %<>% factor(levels=c("teen","young_ad","middle_ad","old_ad")) %>% droplevels()

contrasts(D_FE1.afraid$agegroup) <- contr.sum(3)

dimnames(contrasts(D_FE1.afraid$agegroup))[[2]] <- c("young_ad","middle_ad")

# with random Intercept for ID

Fit0.Video.afraid.r <- lmer(M0.Video.r,D_FE1.afraid,na.action = "na.exclude")

Fit1.Video.afraid.r <- lmer(M1.Video.r,D_FE1.afraid,na.action = "na.exclude")

Fit3.Video.afraid.r <- lmer(M3.Video.r,D_FE1.afraid,na.action = "na.exclude")

# with changed video order

D_FE1.afraid$video %<>% relevel(ref="12") %>% droplevels()

contrasts(D_FE1.afraid$video) <- contr.sum(12)

dimnames(contrasts(D_FE1.afraid$video))[[2]] <- c(12,1:10)

# with random Intercept for ID

Fit1.Video.afraid.r.12 <- lmer(M1.Video.r,D_FE1.afraid,na.action = "na.exclude")

### joyful videos ###

D_FE1.joyful <- D_FE1 %>% filter(emotion=="joyful")

D_FE1.joyful$video %<>% as.factor() %>% droplevels()

contrasts(D_FE1.joyful$video) <- contr.sum(9)

dimnames(contrasts(D_FE1.joyful$video))[[2]] <- c(46:53)

D_FE1.joyful$agegroup %<>% factor(levels=c("teen","young_ad","middle_ad","old_ad"))

contrasts(D_FE1.joyful$agegroup) <- contr.sum(4)

dimnames(contrasts(D_FE1.joyful$agegroup))[[2]] <- c("teen","young_ad","middle_ad")

# with random Intercept for ID

Fit0.Video.joyful.r <- lmer(M0.Video.r,D_FE1.joyful,na.action = "na.exclude")

Fit1.Video.joyful.r <- lmer(M1.Video.r,D_FE1.joyful,na.action = "na.exclude")

Fit3.Video.joyful.r <- lmer(M3.Video.r,D_FE1.joyful,na.action = "na.exclude")

# with changed video order

D_FE1.joyful$video %<>% relevel(ref="54") %>% droplevels()

contrasts(D_FE1.joyful$video) <- contr.sum(9)

dimnames(contrasts(D_FE1.joyful$video))[[2]] <- c(54,46:52)

# with random Intercept for ID

Fit3.Video.joyful.r.54 <- lmer(M3.Video.r,D_FE1.joyful,na.action = "na.exclude")

### sad videos ###

D_FE1.sad <- D_FE1 %>% filter(emotion=="sad")

D_FE1.sad$video %<>% as.factor() %>% droplevels()

contrasts(D_FE1.sad$video) <- contr.sum(14)

dimnames(contrasts(D_FE1.sad$video))[[2]] <- c(34:45,65)

D_FE1.sad$agegroup %<>% factor(levels=c("teen","young_ad","middle_ad","old_ad"))

contrasts(D_FE1.sad$agegroup) <- contr.sum(4)

dimnames(contrasts(D_FE1.sad$agegroup))[[2]] <- c("teen","young_ad","middle_ad")

# with random Intercept for ID

Fit0.Video.sad.r <- lmer(M0.Video.r,D_FE1.sad,na.action = "na.exclude")

Fit1.Video.sad.r <- lmer(M1.Video.r,D_FE1.sad,na.action = "na.exclude")

Fit3.Video.sad.r <- lmer(M3.Video.r,D_FE1.sad,na.action = "na.exclude")

# with changed video order

D_FE1.sad$video %<>% relevel(ref="66") %>% droplevels()

contrasts(D_FE1.sad$video) <- contr.sum(14)

dimnames(contrasts(D_FE1.sad$video))[[2]] <- c(66,34:45)

Fit1.Video.sad.r.65 <- lmer(M1.Video.r,D_FE1.sad,na.action = "na.exclude")

## Model selection

lrtest(Fit0.Video.neutral.r,Fit1.Video.neutral.r,Fit3.Video.neutral.r)

## Likelihood ratio test

##

## Model 1: targetemorating ~ 1 + (1 | ID)

## Model 2: targetemorating ~ 1 + video + (1 | ID)

## Model 3: targetemorating ~ 1 + video + agegroup + agegroup:video + (1 |

## ID)

## #Df LogLik Df Chisq Pr(>Chisq)

## 1 3 -2082.8

## 2 12 -2072.1 9 21.443 0.01082 *

## 3 42 -2066.0 30 12.221 0.99833

## ---

## Signif. codes: 0 '***' 0.001 '**' 0.01 '*' 0.05 '.' 0.1 ' ' 1

ranova(Fit3.Video.neutral.r,test="LRT")

## ANOVA-like table for random-effects: Single term deletions

##

## Model:

## targetemorating ~ video + agegroup + (1 | ID) + video:agegroup

## npar logLik AIC LRT Df Pr(>Chisq)

## <none> 42 -2066.0 4216.0

## (1 | ID) 41 -2186.2 4454.5 240.5 1 < 2.2e-16 ***

## ---

## Signif. codes: 0 '***' 0.001 '**' 0.01 '*' 0.05 '.' 0.1 ' ' 1

lrtest(Fit0.Video.angry.r,Fit1.Video.angry.r,Fit3.Video.angry.r)

## Likelihood ratio test

##

## Model 1: targetemorating ~ 1 + (1 | ID)

## Model 2: targetemorating ~ 1 + video + (1 | ID)

## Model 3: targetemorating ~ 1 + video + agegroup + agegroup:video + (1 |

## ID)

## #Df LogLik Df Chisq Pr(>Chisq)

## 1 3 -1898.5

## 2 12 -1793.3 9 210.45 < 2.2e-16 ***

## 3 38 -1761.5 26 63.54 5.507e-05 ***

## ---

## Signif. codes: 0 '***' 0.001 '**' 0.01 '*' 0.05 '.' 0.1 ' ' 1

ranova(Fit3.Video.angry.r,test="LRT")

## ANOVA-like table for random-effects: Single term deletions

##

## Model:

## targetemorating ~ video + agegroup + (1 | ID) + video:agegroup

## npar logLik AIC LRT Df Pr(>Chisq)

## <none> 38 -1761.5 3599.1

## (1 | ID) 37 -1845.0 3764.0 166.92 1 < 2.2e-16 ***

## ---

## Signif. codes: 0 '***' 0.001 '**' 0.01 '*' 0.05 '.' 0.1 ' ' 1

lrtest(Fit0.Video.disgusted.r,Fit1.Video.disgusted.r,Fit3.Video.disgusted.r)

## Likelihood ratio test

##

## Model 1: targetemorating ~ 1 + (1 | ID)

## Model 2: targetemorating ~ 1 + video + (1 | ID)

## Model 3: targetemorating ~ 1 + video + agegroup + agegroup:video + (1 |

## ID)

## #Df LogLik Df Chisq Pr(>Chisq)

## 1 3 -2079.1

## 2 13 -1967.4 10 223.490 < 2.2e-16 ***

## 3 43 -1941.3 30 52.239 0.007177 **

## ---

## Signif. codes: 0 '***' 0.001 '**' 0.01 '*' 0.05 '.' 0.1 ' ' 1

ranova(Fit3.Video.disgusted.r,test="LRT")

## ANOVA-like table for random-effects: Single term deletions

##

## Model:

## targetemorating ~ video + agegroup + (1 | ID) + video:agegroup

## npar logLik AIC LRT Df Pr(>Chisq)

## <none> 43 -1941.3 3968.5

## (1 | ID) 42 -2091.3 4266.5 300.01 1 < 2.2e-16 ***

## ---

## Signif. codes: 0 '***' 0.001 '**' 0.01 '*' 0.05 '.' 0.1 ' ' 1

lrtest(Fit0.Video.afraid.r,Fit1.Video.afraid.r,Fit3.Video.afraid.r)

## Likelihood ratio test

##

## Model 1: targetemorating ~ 1 + (1 | ID)

## Model 2: targetemorating ~ 1 + video + (1 | ID)

## Model 3: targetemorating ~ 1 + video + agegroup + agegroup:video + (1 |

## ID)

## #Df LogLik Df Chisq Pr(>Chisq)

## 1 3 -1559.0

## 2 14 -1538.9 11 40.065 3.487e-05 ***

## 3 38 -1532.4 24 13.125 0.964

## ---

## Signif. codes: 0 '***' 0.001 '**' 0.01 '*' 0.05 '.' 0.1 ' ' 1

ranova(Fit3.Video.afraid.r,test="LRT")

## ANOVA-like table for random-effects: Single term deletions

##

## Model:

## targetemorating ~ video + agegroup + (1 | ID) + video:agegroup

## npar logLik AIC LRT Df Pr(>Chisq)

## <none> 38 -1532.4 3140.7

## (1 | ID) 37 -1767.4 3608.9 470.12 1 < 2.2e-16 ***

## ---

## Signif. codes: 0 '***' 0.001 '**' 0.01 '*' 0.05 '.' 0.1 ' ' 1

lrtest(Fit0.Video.joyful.r,Fit1.Video.joyful.r,Fit3.Video.joyful.r)

## Likelihood ratio test

##

## Model 1: targetemorating ~ 1 + (1 | ID)

## Model 2: targetemorating ~ 1 + video + (1 | ID)

## Model 3: targetemorating ~ 1 + video + agegroup + agegroup:video + (1 |

## ID)

## #Df LogLik Df Chisq Pr(>Chisq)

## 1 3 -1720.4

## 2 11 -1713.7 8 13.351 0.100313

## 3 38 -1689.1 27 49.262 0.005531 **

## ---

## Signif. codes: 0 '***' 0.001 '**' 0.01 '*' 0.05 '.' 0.1 ' ' 1

ranova(Fit3.Video.joyful.r,test="LRT")

## ANOVA-like table for random-effects: Single term deletions

##

## Model:

## targetemorating ~ video + agegroup + (1 | ID) + video:agegroup

## npar logLik AIC LRT Df Pr(>Chisq)

## <none> 38 -1689.1 3454.2

## (1 | ID) 37 -1782.9 3639.8 187.62 1 < 2.2e-16 ***

## ---

## Signif. codes: 0 '***' 0.001 '**' 0.01 '*' 0.05 '.' 0.1 ' ' 1

lrtest(Fit0.Video.sad.r,Fit1.Video.sad.r,Fit3.Video.sad.r)

## Likelihood ratio test

##

## Model 1: targetemorating ~ 1 + (1 | ID)

## Model 2: targetemorating ~ 1 + video + (1 | ID)

## Model 3: targetemorating ~ 1 + video + agegroup + agegroup:video + (1 |

## ID)

## #Df LogLik Df Chisq Pr(>Chisq)

## 1 3 -2249.0

## 2 16 -2199.6 13 98.856 2.764e-15 ***

## 3 54 -2187.0 38 25.218 0.9445

## ---

## Signif. codes: 0 '***' 0.001 '**' 0.01 '*' 0.05 '.' 0.1 ' ' 1

ranova(Fit3.Video.sad.r,test="LRT")

## ANOVA-like table for random-effects: Single term deletions

##

## Model:

## targetemorating ~ video + agegroup + (1 | ID) + video:agegroup

## npar logLik AIC LRT Df Pr(>Chisq)

## <none> 54 -2187.0 4481.9

## (1 | ID) 53 -2400.2 4906.4 426.44 1 < 2.2e-16 ***

## ---

## Signif. codes: 0 '***' 0.001 '**' 0.01 '*' 0.05 '.' 0.1 ' ' 1

## Results tables for heterogeneity analyses

## Creates an HTML table for the model. Outputs additional information, such as p-values and confidence intervals. ##

tab_model(title = 'Best fitted model for neutral emotion',

dv.labels = c('Model','Model with changed excluded video'),

Fit1.Video.neutral.r, Fit1.Video.neutral.r.64, show.re.var = T,show.icc = T)

| **Best fitted model for neutral emotion** | | | | | | |
| --- | --- | --- | --- | --- | --- | --- |
|  | **Model** | | | **Model with changed excluded video** | | |
| *Predictors* | *Estimates* | *CI* | *p* | *Estimates* | *CI* | *p* |
| (Intercept) | 4.01 | 3.72 – 4.29 | **<0.001** | 4.01 | 3.72 – 4.29 | **<0.001** |
| video [55] | 0.34 | 0.00 – 0.68 | **0.048** | 0.34 | 0.00 – 0.68 | **0.048** |
| video [56] | -0.24 | -0.58 – 0.10 | 0.165 | -0.24 | -0.58 – 0.10 | 0.165 |
| video [57] | -0.37 | -0.71 – -0.03 | **0.032** | -0.37 | -0.71 – -0.03 | **0.032** |
| video [58] | -0.27 | -0.61 – 0.07 | 0.118 | -0.27 | -0.61 – 0.07 | 0.118 |
| video [59] | 0.08 | -0.26 – 0.41 | 0.661 | 0.08 | -0.26 – 0.41 | 0.661 |
| video [60] | -0.34 | -0.68 – -0.00 | **0.049** | -0.34 | -0.68 – -0.00 | **0.049** |
| video [61] | -0.16 | -0.50 – 0.18 | 0.360 | -0.16 | -0.50 – 0.18 | 0.360 |
| video [62] | -0.21 | -0.55 – 0.13 | 0.226 | -0.21 | -0.55 – 0.13 | 0.226 |
| video [63] | 0.66 | 0.32 – 1.00 | **<0.001** |  |  |  |
| video [64] |  |  |  | 0.51 | 0.17 – 0.85 | **0.003** |
| **Random Effects** | | | | | | |
| σ^2^ | 3.25 | | | 3.25 | | |
| τ_00_ | 1.79 _ID_ | | | 1.79 _ID_ | | |
| ICC | 0.36 | | | 0.36 | | |
| N | 99 _ID_ | | | 99 _ID_ | | |
| Observations | 984 | | | 984 | | |
| Marginal R^2^ / Conditional R^2^ | 0.025 / 0.371 | | | 0.025 / 0.371 | | |

tab_model(title = 'Best fitted model for anger emotion',

dv.labels = c('Model','Model with changed excluded video'),

Fit3.Video.angry.r, Fit3.Video.angry.r.22, show.re.var = T,show.icc = T)

| **Best fitted model for anger emotion** | | | | | | | |
| --- | --- | --- | --- | --- | --- | --- | --- |
|  | **Model** | | | **Model with changed excluded video** | | |  |
| *Predictors* | *Estimates* | *CI* | *p* | *Estimates* | *CI* | *p* |  |
| (Intercept) | 3.52 | 2.89 – 4.14 | **<0.001** | 3.52 | 2.89 – 4.14 | **<0.001** |  |
| video [13] | -0.93 | -1.58 – -0.28 | **0.005** | -0.93 | -1.58 – -0.28 | **0.005** |  |
| video [14] | 0.47 | -0.18 – 1.12 | 0.154 | 0.47 | -0.18 – 1.12 | 0.154 |  |
| video [15] | -0.63 | -3.99 – 2.73 | 0.714 | -1.88 | -2.55 – -1.21 | **<0.001** |  |
| video [16] | -0.83 | -1.51 – -0.16 | **0.015** | -0.83 | -1.51 – -0.16 | **0.015** |  |
| video [17] | -0.45 | -1.13 – 0.22 | 0.188 | -0.45 | -1.13 – 0.22 | 0.188 |  |
| video [18] | -0.02 | -0.67 – 0.62 | 0.942 | -0.02 | -0.67 – 0.62 | 0.942 |  |
| video [19] | 1.15 | 0.50 – 1.79 | **0.001** | 1.15 | 0.50 – 1.79 | **0.001** |  |
| video [20] | 0.07 | -0.60 – 0.75 | 0.835 | 0.07 | -0.60 – 0.75 | 0.835 |  |
| video [21] | 1.31 | 0.64 – 1.98 | **<0.001** |  |  |  |  |
| agegroup [teen] | -0.50 | -2.26 – 1.26 | 0.577 | -0.50 | -2.26 – 1.26 | 0.577 |  |
| agegroup [young_ad] | -0.33 | -1.05 – 0.40 | 0.376 | -0.33 | -1.05 – 0.40 | 0.376 |  |
| agegroup [middle_ad] | 0.41 | -0.33 – 1.15 | 0.274 | 0.41 | -0.33 – 1.15 | 0.274 |  |
| video [13] × agegroup [teen] | 0.70 | -1.11 – 2.51 | 0.448 | 0.70 | -1.11 – 2.51 | 0.448 |  |
| video [14] × agegroup [teen] | -0.67 | -2.47 – 1.14 | 0.469 | -0.67 | -2.47 – 1.14 | 0.469 |  |
| video [15] × agegroup [teen] | 4.46 | -5.55 – 14.47 | 0.382 | 0.71 | -0.90 – 2.32 | 0.389 |  |
| video [16] × agegroup [teen] | -0.94 | -2.55 – 0.67 | 0.252 | -0.94 | -2.55 – 0.67 | 0.252 |  |
| video [17] × agegroup [teen] | -1.20 | -2.81 – 0.41 | 0.145 | -1.20 | -2.81 – 0.41 | 0.145 |  |
| video [18] × agegroup [teen] | -0.85 | -2.66 – 0.96 | 0.356 | -0.85 | -2.66 – 0.96 | 0.356 |  |
| video [19] × agegroup [teen] | -0.27 | -2.08 – 1.54 | 0.770 | -0.27 | -2.08 – 1.54 | 0.770 |  |
| video [20] × agegroup [teen] | -0.02 | -1.63 – 1.59 | 0.984 | -0.02 | -1.63 – 1.59 | 0.984 |  |
| video [21] × agegroup [teen] | -0.98 | -2.58 – 0.63 | 0.233 |  |  |  |  |
| video [13] × agegroup [young_ad] | -0.14 | -0.91 – 0.63 | 0.716 | -0.14 | -0.91 – 0.63 | 0.716 |  |
| video [14] × agegroup [young_ad] | 0.57 | -0.20 – 1.35 | 0.146 | 0.57 | -0.20 – 1.35 | 0.146 |  |
| video [15] × agegroup [young_ad] | -1.87 | -5.29 – 1.55 | 0.284 | -0.62 | -1.54 – 0.31 | 0.190 |  |
| video [16] × agegroup [young_ad] | -0.08 | -1.00 – 0.84 | 0.864 | -0.08 | -1.00 – 0.84 | 0.864 |  |
| video [17] × agegroup [young_ad] | 1.04 | 0.12 – 1.96 | **0.027** | 1.04 | 0.12 – 1.96 | **0.027** |  |
| video [18] × agegroup [young_ad] | -0.65 | -1.42 – 0.12 | 0.098 | -0.65 | -1.42 – 0.12 | 0.098 |  |
| video [19] × agegroup [young_ad] | -0.06 | -0.83 – 0.71 | 0.883 | -0.06 | -0.83 – 0.71 | 0.883 |  |
| video [20] × agegroup [young_ad] | 0.14 | -0.78 – 1.06 | 0.766 | 0.14 | -0.78 – 1.06 | 0.766 |  |
| video [21] × agegroup [young_ad] | 0.58 | -0.34 – 1.49 | 0.215 |  |  |  |  |
| video [13] × agegroup [middle_ad] | 0.29 | -0.56 – 1.14 | 0.508 | 0.29 | -0.56 – 1.14 | 0.508 |  |
| video [14] × agegroup [middle_ad] | 0.46 | -0.39 – 1.31 | 0.292 | 0.46 | -0.39 – 1.31 | 0.292 |  |
| video [15] × agegroup [middle_ad] | -1.25 | -4.55 – 2.04 | 0.456 |  |  |  |  |
| video [18] × agegroup [middle_ad] | 0.29 | -0.56 – 1.14 | 0.510 | 0.29 | -0.56 – 1.14 | 0.510 |  |
| video [19] × agegroup [middle_ad] | -0.07 | -0.92 – 0.78 | 0.863 | -0.07 | -0.92 – 0.78 | 0.863 |  |
| video [22] |  |  |  | -0.13 | -0.78 – 0.52 | 0.693 |  |
| video [22] × agegroup [teen] |  |  |  | -0.24 | -2.05 – 1.56 | 0.792 |  |
| video [22] × agegroup [young_ad] |  |  |  | 0.47 | -0.31 – 1.24 | 0.236 |  |
| video [22] × agegroup [middle_ad] |  |  |  | 0.30 | -0.55 – 1.15 | 0.493 |  |
| **Random Effects** | | | | | | | |
| σ^2^ | 2.75 | | | 2.75 | | |  |
| τ_00_ | 1.39 _ID_ | | | 1.39 _ID_ | | |  |
| ICC | 0.34 | | | 0.34 | | |  |
| N | 99 _ID_ | | | 99 _ID_ | | |  |
| Observations | 872 | | | 872 | | |  |
| Marginal R^2^ / Conditional R^2^ | 0.237 / 0.494 | | | 0.237 / 0.494 | | |  |

tab_model(title = 'Best fitted model for disgust emotion',

dv.labels = c('Model','Model with changed excluded video'),

Fit3.Video.disgusted.r, Fit3.Video.disgusted.r.32, show.re.var = T,show.icc = T)

| **Best fitted model for disgust emotion** | | | | | | |
| --- | --- | --- | --- | --- | --- | --- |
|  | **Model** | | | **Model with changed excluded video** | | |
| *Predictors* | *Estimates* | *CI* | *p* | *Estimates* | *CI* | *p* |
| (Intercept) | 4.11 | 3.52 – 4.70 | **<0.001** | 3.99 | 3.40 – 4.58 | **<0.001** |
| video [23] | 0.32 | -3.87 – 4.51 | 0.881 | -0.65 | -4.85 – 3.55 | 0.761 |
| video [24] | 1.44 | 0.81 – 2.06 | **<0.001** | 1.44 | 0.81 – 2.06 | **<0.001** |
| video [25] | 0.86 | 0.26 – 1.46 | **0.005** | 0.99 | 0.38 – 1.59 | **0.001** |
| video [26] | 1.06 | 0.43 – 1.68 | **0.001** | 1.06 | 0.43 – 1.68 | **0.001** |
| video [27] | -0.68 | -1.28 – -0.08 | **0.026** | -0.56 | -1.16 – 0.04 | 0.067 |
| video [28] | -0.32 | -0.92 – 0.28 | 0.302 | -0.19 | -0.80 – 0.41 | 0.525 |
| video [29] | -0.87 | -1.47 – -0.28 | **0.004** | -0.75 | -1.35 – -0.15 | **0.014** |
| video [30] | -0.60 | -1.20 – 0.00 | 0.051 | -0.48 | -1.08 – 0.13 | 0.121 |
| video [31] | 0.98 | 0.38 – 1.58 | **0.001** | 1.10 | 0.48 – 1.73 | **0.001** |
| video [32] | -1.47 | -2.09 – -0.84 | **<0.001** |  |  |  |
| agegroup [teen] | 0.70 | -0.94 – 2.35 | 0.402 | 0.34 | -1.31 – 1.98 | 0.686 |
| agegroup [young_ad] | -0.29 | -0.99 – 0.41 | 0.422 | -0.17 | -0.86 – 0.53 | 0.643 |
| agegroup [middle_ad] | -0.26 | -0.98 – 0.46 | 0.476 | -0.14 | -0.86 – 0.58 | 0.704 |
| video [23] × agegroup [teen] | 2.38 | -10.14 – 14.90 | 0.709 | -0.53 | -13.07 – 12.00 | 0.933 |
| video [24] × agegroup [teen] | 0.69 | -0.80 – 2.18 | 0.364 | 0.69 | -0.80 – 2.18 | 0.364 |
| video [25] × agegroup [teen] | -0.53 | -2.20 – 1.14 | 0.531 | -0.17 | -1.84 – 1.50 | 0.843 |
| video [26] × agegroup [teen] | 0.51 | -0.99 – 2.00 | 0.505 | 0.51 | -0.99 – 2.00 | 0.505 |
| video [27] × agegroup [teen] | -0.27 | -1.94 – 1.40 | 0.749 | 0.09 | -1.58 – 1.76 | 0.914 |
| video [28] × agegroup [teen] | -0.57 | -2.24 – 1.10 | 0.505 | -0.20 | -1.88 – 1.47 | 0.812 |
| video [29] × agegroup [teen] | -1.01 | -2.68 – 0.66 | 0.236 | -0.64 | -2.32 – 1.03 | 0.450 |
| video [30] × agegroup [teen] | 0.11 | -1.56 – 1.78 | 0.900 | 0.47 | -1.20 – 2.14 | 0.581 |
| video [31] × agegroup [teen] | -0.58 | -2.25 – 1.09 | 0.496 | -0.22 | -1.71 – 1.28 | 0.776 |
| video [32] × agegroup [teen] | 0.12 | -1.37 – 1.61 | 0.875 |  |  |  |
| video [23] × agegroup [young_ad] | -1.61 | -5.84 – 2.62 | 0.455 | -0.64 | -4.88 – 3.59 | 0.766 |
| video [24] × agegroup [young_ad] | -0.50 | -1.35 – 0.35 | 0.248 | -0.50 | -1.35 – 0.35 | 0.248 |
| video [25] × agegroup [young_ad] | 0.51 | -0.21 – 1.23 | 0.163 | 0.39 | -0.33 – 1.11 | 0.287 |
| video [26] × agegroup [young_ad] | -0.10 | -0.95 – 0.76 | 0.822 | -0.10 | -0.95 – 0.76 | 0.822 |
| video [27] × agegroup [young_ad] | 0.27 | -0.45 – 0.98 | 0.467 | 0.14 | -0.57 – 0.86 | 0.693 |
| video [28] × agegroup [young_ad] | 0.15 | -0.57 – 0.87 | 0.683 | 0.03 | -0.69 – 0.75 | 0.939 |
| video [29] × agegroup [young_ad] | -0.27 | -0.98 – 0.45 | 0.462 | -0.39 | -1.10 – 0.32 | 0.285 |
| video [30] × agegroup [young_ad] | 0.18 | -0.54 – 0.90 | 0.622 | 0.06 | -0.66 – 0.78 | 0.872 |
| video [31] × agegroup [young_ad] | 0.43 | -0.28 – 1.15 | 0.235 | 0.31 | -0.54 – 1.17 | 0.473 |
| video [32] × agegroup [young_ad] | 0.93 | 0.08 – 1.77 | **0.033** |  |  |  |
| video [23] × agegroup [middle_ad] | -0.93 | -5.08 – 3.21 | 0.659 | 0.04 | -4.11 – 4.19 | 0.985 |
| video [25] × agegroup [middle_ad] | 0.24 | -0.54 – 1.02 | 0.549 | 0.12 | -0.67 – 0.90 | 0.767 |
| video [27] × agegroup [middle_ad] | -0.40 | -1.19 – 0.38 | 0.312 | -0.53 | -1.31 – 0.26 | 0.189 |
| video [28] × agegroup [middle_ad] | 0.13 | -0.65 – 0.92 | 0.737 | 0.01 | -0.77 – 0.80 | 0.974 |
| video [29] × agegroup [middle_ad] | 1.07 | 0.29 – 1.86 | **0.007** | 0.95 | 0.17 – 1.74 | **0.017** |
| video [30] × agegroup [middle_ad] | -0.25 | -1.03 – 0.53 | 0.529 | -0.37 | -1.16 – 0.41 | 0.351 |
| video [31] × agegroup [middle_ad] | 0.12 | -0.66 – 0.90 | 0.761 |  |  |  |
| video [33] |  |  |  | -1.35 | -1.95 – -0.75 | **<0.001** |
| video [33] × agegroup [teen] |  |  |  | 0.48 | -1.19 – 2.16 | 0.570 |
| video [33] × agegroup [young_ad] |  |  |  | 0.80 | 0.09 – 1.52 | **0.027** |
| video [33] × agegroup [middle_ad] |  |  |  | -0.12 | -0.90 – 0.66 | 0.761 |
| **Random Effects** | | | | | | |
| σ^2^ | 2.34 | | | 2.34 | | |
| τ_00_ | 1.58 _ID_ | | | 1.58 _ID_ | | |
| ICC | 0.40 | | | 0.40 | | |
| N | 99 _ID_ | | | 99 _ID_ | | |
| Observations | 996 | | | 996 | | |
| Marginal R^2^ / Conditional R^2^ | 0.184 / 0.512 | | | 0.184 / 0.512 | | |

tab_model(title = 'Best fitted model for fear emotion',

dv.labels = c('Model','Model with changed excluded video'),

Fit1.Video.afraid.r, Fit1.Video.afraid.r.12, show.re.var = T,show.icc = T)

| **Best fitted model for fear emotion** | | | | | | |
| --- | --- | --- | --- | --- | --- | --- |
|  | **Model** | | | **Model with changed excluded video** | | |
| *Predictors* | *Estimates* | *CI* | *p* | *Estimates* | *CI* | *p* |
| (Intercept) | 3.37 | 3.00 – 3.73 | **<0.001** | 3.37 | 3.00 – 3.73 | **<0.001** |
| video [1] | -0.45 | -0.75 – -0.16 | **0.003** | -0.45 | -0.75 – -0.16 | **0.003** |
| video [2] | 0.14 | -0.16 – 0.44 | 0.359 | 0.14 | -0.16 – 0.44 | 0.359 |
| video [3] | -0.03 | -0.33 – 0.26 | 0.829 | -0.03 | -0.33 – 0.26 | 0.829 |
| video [4] | 0.24 | -0.06 – 0.54 | 0.115 | 0.24 | -0.06 – 0.54 | 0.115 |
| video [5] | -0.02 | -0.32 – 0.28 | 0.904 | -0.02 | -0.32 – 0.28 | 0.904 |
| video [6] | 0.24 | -0.06 – 0.53 | 0.115 | 0.24 | -0.06 – 0.53 | 0.115 |
| video [7] | 0.18 | -0.12 – 0.48 | 0.230 | 0.18 | -0.12 – 0.48 | 0.230 |
| video [8] | 0.65 | 0.36 – 0.95 | **<0.001** | 0.65 | 0.36 – 0.95 | **<0.001** |
| video [9] | -0.65 | -0.94 – -0.35 | **<0.001** | -0.65 | -0.94 – -0.35 | **<0.001** |
| video [10] | 0.19 | -0.10 – 0.49 | 0.195 | 0.19 | -0.10 – 0.49 | 0.195 |
| video [11] | -0.59 | -0.89 – -0.29 | **<0.001** |  |  |  |
| video [12] |  |  |  | 0.10 | -0.20 – 0.39 | 0.522 |
| **Random Effects** | | | | | | |
| σ^2^ | 1.75 | | | 1.75 | | |
| τ_00_ | 2.29 _ID_ | | | 2.29 _ID_ | | |
| ICC | 0.57 | | | 0.57 | | |
| N | 71 _ID_ | | | 71 _ID_ | | |
| Observations | 844 | | | 844 | | |
| Marginal R^2^ / Conditional R^2^ | 0.032 / 0.582 | | | 0.032 / 0.582 | | |

tab_model(title = 'Best fitted model for happiness emotion',

dv.labels = c('Model','Model with changed excluded video'),

Fit3.Video.joyful.r, Fit3.Video.joyful.r.54, show.re.var = T,show.icc = T)

| **Best fitted model for happiness emotion** | | | | | | |
| --- | --- | --- | --- | --- | --- | --- |
|  | **Model** | | | **Model with changed excluded video** | | |
| *Predictors* | *Estimates* | *CI* | *p* | *Estimates* | *CI* | *p* |
| (Intercept) | 3.63 | 3.40 – 3.86 | **<0.001** | 3.63 | 3.40 – 3.86 | **<0.001** |
| video [46] | -0.06 | -0.34 – 0.21 | 0.661 | -0.06 | -0.34 – 0.21 | 0.661 |
| video [47] | 0.07 | -0.21 – 0.35 | 0.618 | 0.07 | -0.21 – 0.35 | 0.618 |
| video [48] | -0.28 | -0.56 – -0.01 | **0.045** | -0.28 | -0.56 – -0.01 | **0.045** |
| video [49] | 0.44 | 0.17 – 0.72 | **0.002** | 0.44 | 0.17 – 0.72 | **0.002** |
| video [50] | 0.30 | 0.02 – 0.57 | **0.037** | 0.30 | 0.02 – 0.57 | **0.037** |
| video [51] | -0.12 | -0.39 – 0.16 | 0.411 | -0.12 | -0.39 – 0.16 | 0.411 |
| video [52] | -0.11 | -0.39 – 0.16 | 0.419 | -0.11 | -0.39 – 0.16 | 0.419 |
| video [53] | 0.28 | 0.00 – 0.55 | **0.049** |  |  |  |
| agegroup [teen] | -0.22 | -0.60 – 0.16 | 0.262 | -0.22 | -0.60 – 0.16 | 0.262 |
| agegroup [young_ad] | -0.60 | -1.00 – -0.21 | **0.003** | -0.60 | -1.00 – -0.21 | **0.003** |
| agegroup [middle_ad] | 0.11 | -0.31 – 0.53 | 0.619 | 0.11 | -0.31 – 0.53 | 0.619 |
| video [46] × agegroup [teen] | 0.47 | 0.01 – 0.92 | **0.045** | 0.47 | 0.01 – 0.92 | **0.045** |
| video [47] × agegroup [teen] | -0.38 | -0.84 – 0.08 | 0.103 | -0.38 | -0.84 – 0.08 | 0.103 |
| video [48] × agegroup [teen] | 0.54 | 0.09 – 1.00 | **0.020** | 0.54 | 0.09 – 1.00 | **0.020** |
| video [49] × agegroup [teen] | -0.15 | -0.60 – 0.31 | 0.532 | -0.15 | -0.60 – 0.31 | 0.532 |
| video [50] × agegroup [teen] | -0.28 | -0.74 – 0.17 | 0.224 | -0.28 | -0.74 – 0.17 | 0.224 |
| video [51] × agegroup [teen] | 0.02 | -0.44 – 0.48 | 0.932 | 0.02 | -0.44 – 0.48 | 0.932 |
| video [52] × agegroup [teen] | -0.09 | -0.54 – 0.37 | 0.702 | -0.09 | -0.54 – 0.37 | 0.702 |
| video [53] × agegroup [teen] | 0.59 | 0.14 – 1.05 | **0.011** |  |  |  |
| video [46] × agegroup [young_ad] | 0.15 | -0.32 – 0.62 | 0.532 | 0.15 | -0.32 – 0.62 | 0.532 |
| video [47] × agegroup [young_ad] | -0.20 | -0.68 – 0.28 | 0.420 | -0.20 | -0.68 – 0.28 | 0.420 |
| video [48] × agegroup [young_ad] | 0.16 | -0.32 – 0.63 | 0.526 | 0.16 | -0.32 – 0.63 | 0.526 |
| video [49] × agegroup [young_ad] | -0.40 | -0.88 – 0.08 | 0.100 | -0.40 | -0.88 – 0.08 | 0.100 |
| video [50] × agegroup [young_ad] | 0.08 | -0.40 – 0.56 | 0.749 | 0.08 | -0.40 – 0.56 | 0.749 |
| video [51] × agegroup [young_ad] | -0.24 | -0.71 – 0.24 | 0.329 | -0.24 | -0.71 – 0.24 | 0.329 |
| video [52] × agegroup [young_ad] | 0.07 | -0.41 – 0.55 | 0.774 | 0.07 | -0.41 – 0.55 | 0.774 |
| video [53] × agegroup [young_ad] | 0.45 | -0.02 – 0.93 | 0.061 |  |  |  |
| video [46] × agegroup [middle_ad] | -0.49 | -0.99 – 0.01 | 0.056 | -0.49 | -0.99 – 0.01 | 0.056 |
| video [47] × agegroup [middle_ad] | 0.24 | -0.27 – 0.74 | 0.355 | 0.24 | -0.27 – 0.74 | 0.355 |
| video [48] × agegroup [middle_ad] | -0.03 | -0.53 – 0.47 | 0.905 | -0.03 | -0.53 – 0.47 | 0.905 |
| video [49] × agegroup [middle_ad] | 0.34 | -0.16 – 0.84 | 0.184 | 0.34 | -0.16 – 0.84 | 0.184 |
| video [50] × agegroup [middle_ad] | -0.18 | -0.68 – 0.32 | 0.485 | -0.18 | -0.68 – 0.32 | 0.485 |
| video [51] × agegroup [middle_ad] | -0.15 | -0.65 – 0.35 | 0.558 | -0.15 | -0.65 – 0.35 | 0.558 |
| video [52] × agegroup [middle_ad] | 0.13 | -0.37 – 0.64 | 0.598 | 0.13 | -0.37 – 0.64 | 0.598 |
| video [53] × agegroup [middle_ad] | 0.22 | -0.28 – 0.72 | 0.387 |  |  |  |
| video [54] |  |  |  | -0.51 | -0.79 – -0.24 | **<0.001** |
| video [54] × agegroup [teen] |  |  |  | -0.73 | -1.18 – -0.27 | **0.002** |
| video [54] × agegroup [young_ad] |  |  |  | -0.07 | -0.55 – 0.41 | 0.767 |
| video [54] × agegroup [middle_ad] |  |  |  | -0.09 | -0.59 – 0.42 | 0.739 |
| **Random Effects** | | | | | | |
| σ^2^ | 2.16 | | | 2.16 | | |
| τ_00_ | 1.10 _ID_ | | | 1.10 _ID_ | | |
| ICC | 0.34 | | | 0.34 | | |
| N | 99 _ID_ | | | 99 _ID_ | | |
| Observations | 884 | | | 884 | | |
| Marginal R^2^ / Conditional R^2^ | 0.130 / 0.424 | | | 0.130 / 0.424 | | |

tab_model(title = 'Best fitted model for sadness emotion',

dv.labels = c('Model','Model with changed excluded video'),

Fit1.Video.sad.r, Fit1.Video.sad.r.65, show.re.var = T,show.icc = T)

| **Best fitted model for sadness emotion** | | | | | | |
| --- | --- | --- | --- | --- | --- | --- |
|  | **Model** | | | **Model with changed excluded video** | | |
| *Predictors* | *Estimates* | *CI* | *p* | *Estimates* | *CI* | *p* |
| (Intercept) | 4.33 | 4.10 – 4.56 | **<0.001** | 4.33 | 4.10 – 4.56 | **<0.001** |
| video [34] | 0.50 | 0.22 – 0.78 | **<0.001** | 0.50 | 0.22 – 0.78 | **<0.001** |
| video [35] | -0.28 | -0.52 – -0.04 | **0.023** | -0.28 | -0.52 – -0.04 | **0.023** |
| video [36] | 0.29 | 0.05 – 0.53 | **0.017** | 0.29 | 0.05 – 0.53 | **0.017** |
| video [37] | 0.35 | 0.11 – 0.59 | **0.004** | 0.35 | 0.11 – 0.59 | **0.004** |
| video [38] | 0.54 | 0.30 – 0.78 | **<0.001** | 0.54 | 0.30 – 0.78 | **<0.001** |
| video [39] | -0.04 | -0.28 – 0.20 | 0.716 | -0.04 | -0.28 – 0.20 | 0.716 |
| video [40] | 0.30 | 0.06 – 0.54 | **0.014** | 0.30 | 0.06 – 0.54 | **0.014** |
| video [41] | 0.51 | 0.27 – 0.75 | **<0.001** | 0.51 | 0.27 – 0.75 | **<0.001** |
| video [42] | -1.00 | -1.28 – -0.72 | **<0.001** | -1.00 | -1.28 – -0.72 | **<0.001** |
| video [43] | -0.17 | -0.45 – 0.11 | 0.237 | -0.17 | -0.45 – 0.11 | 0.237 |
| video [44] | 0.01 | -0.23 – 0.25 | 0.958 | 0.01 | -0.23 – 0.25 | 0.958 |
| video [45] | -0.54 | -0.82 – -0.25 | **<0.001** | -0.54 | -0.82 – -0.25 | **<0.001** |
| video [65] | -0.10 | -0.34 – 0.14 | 0.435 |  |  |  |
| video [66] |  |  |  | -0.38 | -0.62 – -0.14 | **0.002** |
| **Random Effects** | | | | | | |
| σ^2^ | 1.56 | | | 1.56 | | |
| τ_00_ | 1.19 _ID_ | | | 1.19 _ID_ | | |
| ICC | 0.43 | | | 0.43 | | |
| N | 99 _ID_ | | | 99 _ID_ | | |
| Observations | 1263 | | | 1263 | | |
| Marginal R^2^ / Conditional R^2^ | 0.058 / 0.466 | | | 0.058 / 0.466 | | |
